# A symmetry model for genetic coding via a wallpaper group composed of the traditional four bases and an imaginary base E: Towards category theory-like systematization of molecular/genetic biology

**DOI:** 10.1186/1742-4682-11-18

**Published:** 2014-05-07

**Authors:** Jitsuki Sawamura, Shigeru Morishita, Jun Ishigooka

**Affiliations:** 1 Department of Psychiatry, Tokyo Women’s Medical University, Tokyo, Japan; 2 Depression Prevention Medical Center, Inariyama Takeda Hospital, Kyoto, Japan

**Keywords:** DNA (RNA) bases, Imaginary base, Wallpaper group, Operation, Cartesian vector, Category, Central dogma

## Abstract

**Background:**

Previously, we suggested prototypal models that describe some clinical states based on group postulates. Here, we demonstrate a group/category theory-like model for molecular/genetic biology as an alternative application of our previous model. Specifically, we focus on deoxyribonucleic acid (DNA) base sequences.

**Results:**

We construct a wallpaper pattern based on a five-letter cruciform motif with letters C, A, T, G, and E. Whereas the first four letters represent the standard DNA bases, the fifth is introduced for ease in formulating group operations that reproduce insertions and deletions of DNA base sequences. A basic group Z_5_ = {r, u, d, l, n} of operations is defined for the wallpaper pattern, with which a sequence of points can be generated corresponding to changes of a base in a DNA sequence by following the orbit of a point of the pattern under operations in group Z_5_. Other manipulations of DNA sequence can be treated using a vector-like notation ‘D_j_’ corresponding to a DNA sequence but based on the five-letter base set; also, ‘D_j_’s are expressed graphically. Insertions and deletions of a series of letters ‘E’ are admitted to assist in describing DNA recombination. Likewise, a vector-like notation R_j_ can be constructed for sequences of ribonucleic acid (RNA). The wallpaper group B = {Z_5_^×∞^, ●} (an ∞-fold Cartesian product of Z_5_) acts on D_j_ (or R_j_) yielding changes to D_j_ (or R_j_) denoted by ‘D_j_◦B_(j→k)_ = D_k_’ (or ‘R_j_◦B_(j→k)_ = R_k_’). Based on the operations of this group, two types of groups—a modulo 5 linear group and a rotational group over the Gaussian plane, acting on the five bases—are linked as parts of the wallpaper group for broader applications. As a result, changes, insertions/deletions and DNA (RNA) recombination (partial/total conversion) are described. As an exploratory study, a notation for the canonical “central dogma” via a category theory-like way is presented for future developments.

**Conclusions:**

Despite the large incompleteness of our methodology, there is fertile ground to consider a symmetry model for genetic coding based on our specific wallpaper group. A more integrated formulation containing “central dogma” for future molecular/genetic biology remains to be explored.

## Background

Group theory is the cornerstone in classifying and studying abstract concepts involving symmetry [1,2]. In general, when group theory is used in various fields of natural sciences, it plays an important role in describing geometrical or dynamical symmetries of phenomena under consideration; examples include mathematics [3,4], physics [5-8], chemistry [9], molecular/genetic biology [10-22], and anthropology [23]. Moreover, much fertile ground still exists where group theory can display its versatility from a multitude of viewpoints. To our knowledge, one such candidate is molecular/genetic biology where group theory has already provided great contributions [10-22].

Deoxyribonucleic acid (DNA) is a nucleic acid containing genetic instructions coded in ordered sequences of four bases located in genes that determine specific genetic characteristics of an organism. In the canonical Watson-Crick DNA base pairing, adenine (A) forms a base pair with thymine (T) and guanine (G) forms a base pair with cytosine (C) [24-26]. Similarly, ribonucleic acid (RNA), which has various biological roles, is a molecule that has a much shorter chain of nucleotides. The sequence of DNA consisting of bases ‘A, C, T and G’ is transcribed into RNA, composed of bases ‘A, C, U and G’; the sets differ in that ‘U (uracil)’ replaces ‘T (thymine)’.

Over the latter half of the 20th century, the nature of the genetic code became fairly well established. As for the coding sequences of DNA into nucleotide units, one needs to build up more general, sophisticated, rationally functionalized systematics concerning DNA base sequences that will enable genes to be understood at the molecular biology level in more optimized form. Indeed, many approaches have been undertaken to describe gene characteristics from various viewpoints within the participating disciplines [24-42]. In particular, the concept of ‘symmetry’ for DNA sequences plays an important role in understanding their characteristics.

However, each has its advantages and disadvantages in terms of utility and convenience in applications. To our knowledge, so far, if we intend to incorporate a sequence of bases into another sequence and/or exclude certain bases from that substitution, we need to look further afield because normally, sequencing and inserting-deleting operations cannot help in distinguishing one from the other. That means that multiple types of operations are necessary if features of DNA containing exceptional sequences are to be treated.

Previously, we suggested prototypal models that describe some clinical states based on group postulates [43]. In this article, we demonstrate a group/category theory-like model for molecular/genetic biology as an alternative application of our previous model. Specifically, focusing on DNA base sequences, we present a simple model where not only changes in sequences of DNA bases but also insertion, deletion, and recombination (partial/total conversion) of DNA bases are treatable within some simple rules via the combination of a set and a group defined over some specific wallpaper pattern. Moreover, a category theory-like formalism, where a description of the DNA bases and their transcription to RNA bases can be made, is attempted from which a category theory-like framework is constructed requiring as few and as simple rules as possible. As an example, by assimilating the canonical “central dogma” [26], we hope to provoke more interactivity among those interested branches of natural science, if possible. The methodology consists of eight parts, the content of which is built-up step-by-step as scope is enlarged to encompass the more advanced themes.

## §1 A preliminary setting describing a wallpaper pattern used as a symmetry model for DNA sequences

**Figure 1:**
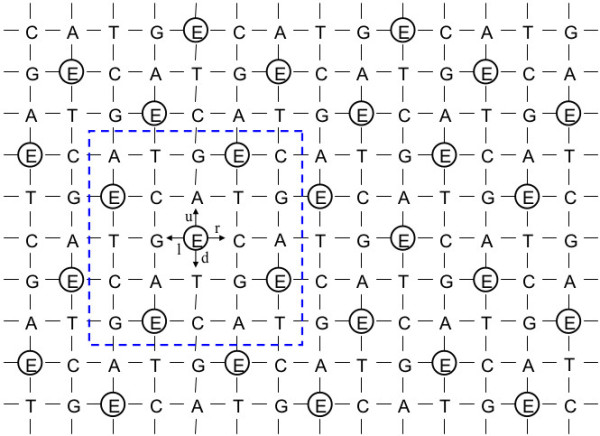
**Wallpaper pattern using the five bases.** A point ‘P’ is assumed to move step-by-step over the wallpaper-like grid-point array where four DNA bases ‘C, A, T, G’ and imaginary ‘E’ forming the cruciform motif is used to generate the pattern. The unit cell enclosed by the blue dashed line can also be used to establish the pattern.

First, we consider a certain wallpaper pattern that helps us to visualize the operations of the present model (see Figure 1) [2,44-47]. There, the pattern comprises repetitions of a cruciform motif with each motif consisting of five letters E, C, A, T, and G with the latter four letters equally spaced at the points of a cross about a central E. The motif generates the pattern through a translation specified as a knight’s move in chess—two steps out and one step right. In this way, the grid-points in this regular wallpaper pattern can be obtained uniquely and be extended indefinitely. Note also that each horizontal line is generated by repetitions of the sequence E-C-A-T-G. Moreover, the line above is a displaced copy of the one below with letter A placed directly above letter E. This preserves the condition that any cruciform is composed of one each of the five letters.

The wallpaper pattern as an array of cruciforms is capable of being constructed as stacks of a unit cell (the 5 × 5 square enclosed in the dotted line in Figure 1) by horizontal and vertical translations [2,44-47]. The positions of the bases of the cruciform motif are so determined to make it easier to determine the complementary base of each base; the practical applications are clarified later. We introduce the letter ‘E’ to indicate an ‘empty’ base which is treated in the same way as the other bases at least for display purposes. This five-base scheme is adopted to aid the notion of group composition in our model.In addition, we focus on a point ‘P’ on the wallpaper pattern (i.e., the grid-point array in Figure 1), to compose a certain DNA base sequence. In accordance with this, we shall always adjoin a series of letters that are determined as a trajectory of the point ‘P’—also called the ‘orbit of P’—over the wallpaper pattern. For instance, when we identify or recognize some changes of DNA bases with ‘P’ moving from ‘A → C → E’ over the wallpaper pattern, then this represents a series of changes to one base located at a specific position of a DNA sequence in the manner ‘ACE…’ or ‘…A…’ → ‘…C…’ → ‘…E…’. The ‘orbit of P’ can describe series of sequences of DNA bases, or series of changes of each letters in the same places, although, in this article, we focus mainly on the latter case, without provisory context.

**Figure 2:**
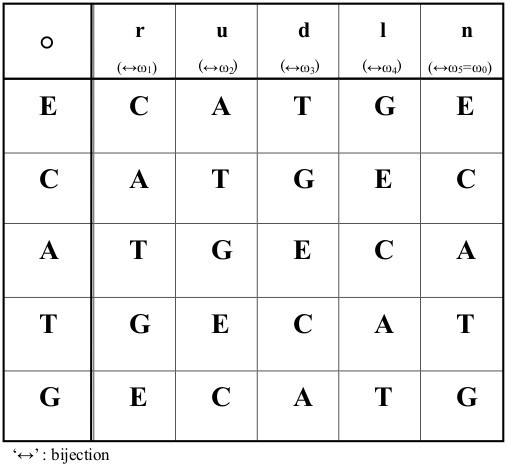
**Cayley tables for the five bases and five operations of linear/rotational groups.** Any of the five operations on any of five bases yields a base in a cyclic order.

**Figure 3:**
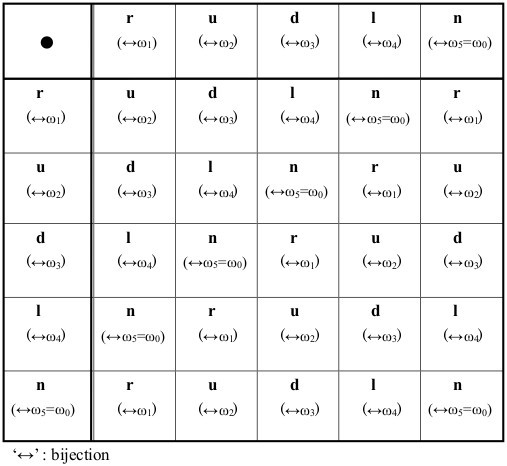
**Cayley tables for the linear group, rotational group and wallpaper group for five bases.** This confirms the bijection between the wallpaper group and rotational group.

With these postulates, we consider the set C_5_ = {C, A, T, G, E}. If the point ‘P’ moves onto an ‘E’, ‘E’ must be included and identified in the series of letters, as in ‘ACG**E**T’, for example. This is interpreted as the series of DNA bases ‘ACGT’. Thus, ‘E’ depends on context; that is, ‘E’ can be inserted or removed from any series where we would like to include or eliminate ‘E’s so long as these are recognized/tracked in the entire process. When read from left to right, the place number of each letter in the series is subscripted, as in ‘A_1_C_2_G_3_T_4_’. After insertions/deletions, the place number is augmented/diminished depending on initial and final positions; hence following three insertions ‘A_1_C_2_G_3_T_4_’ → ‘A_1_**E**_**2**_C_3_G_4_**E**_**5**_**E**_**6**_T_7_’; this means the point ‘P’ takes the place ‘E’ once between A_1_ and C_3_, and twice between G_4_ and T_7_ over the wallpaper pattern in Figure 1. More details are to be given later.As a further refinement, the orbit of ‘P’ can be stated as a sequence of shift operations as follows; let ‘r’ denote a move one step to the right corresponding to say A → T, T → G or G → E. Similarly, we denote ‘l’: move one step to the left as for C → E, and E → G; ‘u’: move one step up; and ‘d’: move down. We include ‘n’ to designate a ‘no move’ (remain at the same point). A sequence of ‘r’, ‘u’, ‘d’, ‘l’, and ‘n’ then provides a position-independent means to describe the orbit of ‘P’; any of these five operations can be applied to any of the five letters. We denote their operations on ‘P’ in the following way. If point ‘P’ moves from ‘E’ to ‘C’ (step to the right), we write ‘E◦r = C’ where ‘◦’ signifies apply ‘r’ to ‘E’ (see Figure 1). In a similar way, ‘E◦l = G’, ‘E◦u = A’, ‘E◦d = T’ and ‘E◦n = E’. Note though that each operator means a change of one base to another base within these five bases; the meaning of ‘=’ is not the degree of translation but equivalence to the resultant base from the wallpaper pattern.To shorten multiple applications of the operations, we introduce ‘●’ to denote the composition of two operations, for example, ‘((E◦r)◦u) = E◦r●u’. From Figure 1, we find ‘E◦d = T’ yields the same change as ‘E◦r●u = T’. As other examples, ‘r●r●d = n’ results in ‘r●r = u’, and ‘d●d●l = n’ results in ‘d●d = r’, because from Figure 1, ‘E◦r●r = E◦u = A’, and ‘E◦d●d = E◦r = C. All possible one-step changes between letters ‘C, A, T, G, E’ and operators ‘r, u, d, l, n’, and all possible compositions of operators for the wallpaper pattern of Figure 1 are presented in Figures 2 and 3, and Appendix A.

The binary compositions among the five operations ‘r, u, d, l and n’ can be shown to satisfy the Abelian group postulates (wallpaper group/plane symmetry group/plane crystallographic group [2-4,44-47]). Indeed, let Z_5_ = {r, u, d, l, n}, then {Z_5_, ●} is the Abelian group of order five. That is, for all elements ∈ Z_5_, we have:

1) Associativity: x●(y●z) = (x●y)●z, (x, y or z being arbitrary elements belonging to Z_5_);

2) Identity: ‘n’ is an identity element such that x●n = n●x = x;

3) Inverse: a unique element x^−1^ exists such that x● x^−1^ = x^−1^●x = n (x^−1^ is called the inverse element of x);

4) Commutativity: x●y = y●x,

5) Closure: any combination of operations between x●y belongs to Z_5_.

Therefore, Z_5_ is an Abelian group [2-4,44-47]. The inverses for each of the elements are:

(1) $$r^{-1}=l,l^{-1}=r,u^{-1}=d,d^{-1}=u,n^{-1}=n,$$

which can be used to complete the composition table—also known as the Cayley table of the group.

We further stipulate that when we perform these operations, then we always assume/identify the coding of the sequence of DNA bases in accordance with these operations, and vice versa. This is because the action of ‘u’ on E yields base ‘A’, that of ‘d’ on ‘E’ yields base ‘T’, that of ‘l’ on ‘E’ yield base ‘G’, and naturally that of ‘n’ on ‘E’ results in the same ‘E’. For a more complex example, we might insert a certain series of ‘A’s in ‘ACCGT’ between the 3rd and 4th base. To begin, we decide to write this manipulation as follows: ‘ACC( )GT’ is transformed into ‘ACC(**E**)GT’ by inserting ‘E’. Next, because the operation ‘u’ to the new 4th component ‘E’ yields ‘E → A’ (‘ACCGT’ → ‘ACC**A**GT’), and vice versa, that is, ‘d’ operating on the 4th component ‘A’ produces ‘A → E’ (‘ACC**A**GT’ → ‘ACCGT’). In this way, appropriate use of ‘E’s through the adequate combination of operators of Z_5_ enables to express inclusion and/or exclusion of any base between bases in a DNA sequence. To indicate this, we adopt a vector-like description with an infinite number of ‘E’s being assumed to be present at the end of any given base sequence. This means the point ‘P’ takes ‘E’s an infinite number of times over the wallpaper pattern (Figure 1); i.e.,

(2a) $$\begin{array}{l} D_{j}=\left[C\left|T\right|G\left|A\right|T\left|A\right|A\left|C\right|E\left|E\right|E\left|E\right|E\left|E\right|\ldots \right]\\ \;=\left[C_{1}|T_{2}\left(\;\right)G_{3}\left|A_{4}\right|T_{5}\left(\;\right)A_{6}\left|A_{7}\right|C_{8}\left|E_{9}\right|E_{10}\left|E_{11}\right|E_{12}\left|E_{13}\right|E_{14}|\ldots \right] \end{array}$$

(2b) $$\;=\left[C_{1}|T_{2}\left(E_{3}\right)G_{4}\left|A_{5}\right|T_{6}\left(E_{7}|E_{8}\right)A_{9}\left|A_{10}\right|C_{11}\left|E_{12}\right|E_{13}\left|E_{14}\right|E_{15}\left|E_{16}\right|\ldots \right].$$

(j: the number of the sequence, N: the number of single-stranded DNA bases of ‘D_j_’s except for the infinite tail of ‘E’s; in the above case, N = 8)

In the last expression (2b), ‘E_3_’ is inserted before the 3rd component ‘G_3_’ and 6th component ‘A_6_’ marked by ‘( )’ in formula (2a), and the place numbers of all components to the right of the 3rd component are all incremented by ‘1’, those to the right of the 6th component; by ‘1 + 2’. Likewise, we assume that the deletion of any ‘E’s that are already displayed in D_j_ is always permissible according to need with the place numbers being decreased by the necessary size.

Essentially, we regard the subscripted place number of a component of D_j_, e.g., ‘**3**’ of ‘A_**3**_’, as a convenient place mark to help in recognizing and counting the order of sequences. Place numbers remain fixed when performing operations within a series of operations during code recognition of bases. However, for an operation, another place number is always permissible in principle, from where indexing of a specific DNA base sequence starts.

Alternatively, we use the following notation to describe various cases:

1) we denote by ‘{D_j_}’ a sequence ‘D_j_’ where specified ‘E’s other than the trailing series of ‘E’s are implicitly implied but the place number indexing is retained; i.e.,

(3a) $$\left\{D_{j}\right\}=\left[C_{1}\left|T_{2}\right|G_{4}\left|A_{5}\right|T_{6}\left|A_{9}\right|A_{10}\left|C_{11}\right|E_{12}\left|E_{13}\right|E_{14}\left|E_{15}\right|E_{16}|\ldots \right].$$

Here, the explicitly indicated place numbers are the same as in (2b) and missing subscripted place numbers indicate omitted ‘E’s. Hence, (3a) without trailing ‘E’s and subscripts represents an ordinal/conventional DNA sequence.

2) we denote by ‘<D_j_>’ a sequence ‘D_j_’ where specified explicit ‘E’s other than the trailing ‘E’s are deleted (changed into implicit ‘E’s) and the base sequence is re-indexed with sequential place numbers, i.e.,

(3b) $$<D_{j}>=\left[C_{1}\left|T_{2}\right|G_{3}\left|A_{4}\right|T_{5}\left|A_{6}\right|A_{7}\left|C_{8}\right|E_{9}\left|E_{10}\right|E_{11}\left|E_{12}\right|E_{13}|\ldots \right].$$

Note that ‘E’s other than the trailing ‘E’s are not recognized as explicit components and hence are not indexed. Additional insertions/deletions of ‘E’s are permitted after deletions of ‘E’s; therefore, apart from the trailing ‘E’s, (3b) signifies an ordinal/conventional DNA sequence.

Although equivalent to ‘CTGATAAC’ as an actual DNA sequence expressions, related expressions {D_j_} and <D_j_> differ from each other; the former retains all information regarding inserted ‘E’s and place numbers whereas the latter does not.

In an extension of the notation, a multiple sequence of deletions of ‘E’s (say t-times) can be written as a t-tuple of ‘< >’s denoted ‘<<<<D_j_>>>> (t-tuple) = <D_j_> _t_’. The final expression is without explicit ‘E’s other than those trailing at the end, and thus formulates a genuine DNA sequence after the appearance of indels. (Short for insertion/deletion markers, the idels are strings of mutated base pairs.) Similarly for the operation { }, we have ‘{{{D_j_}}} (t-tuple) = {D_j_}_t_’. The operations ‘{ }’ and ‘< >’ can be performed freely when necessary; if further indels occur at say ‘G_3_’ and ‘A_7_’ in

<D_j_ > = [C_1_|T_2_|G_3_|A_4_|T_5_|A_6_|A_7_|C_8_|**E**_9_|**E**_10_|**E**_11_|**E**_12_|**E**_13_|…], then < D_j_ > changes into

<D_j1_ > = [C_1_|T_2_(**E**_**3**_)A_4_|T_5_|A_6_(**E**_**7**_)C_8_|**E**_9_|**E**_10_|**E**_11_|**E**_12_|**E**_13_|…], and subsequently into

<<D_j1_>> = [C_1_|T_2_|A_3_|T_4_|A_5_|C_6_|**E**_7_|**E**_8_|**E**_9_|**E**_10_|**E**_11_|…]. The sequence < D_j1_ > contains implicit ‘E’s aside from the trailing ‘E’s, and can be written as

{<D_j1_>} = [C_1_|T_2_|A_4_|T_5_|A_6_|C_8_|**E**_9_|**E**_10_|**E**_11_|**E**_12_|**E**_13_|…]. Naturally, {<D_j1_>} and < D_j1_ > are equivalent, but < <D_j1_> > and < D_j1_ > differ. Moreover, as long as place numbers are recognized/traced precisely, combinations of manipulations ‘{ }’ and ‘< >’ are allowed; e.g., {<{{<D_j1_>}}>}. Hence, with appropriate use, we could treat (read, interpret, describe, record) conventional sequences of DNA via ‘{D_j_}’ or ‘<D_j_>’. However, below we shall focus on simple sequences ‘D_j_’.

Looking at the beginning of a base sequence as in the following:

$$D_{j}=\left[C_{1}\left|G_{2}\right|A_{3}\left|C_{4}\right|\ldots \left|T_{i}\right|\ldots \left|A_{N-1}\right|T_{N}\left|E_{N+1}\right|E_{N+2}\left|E_{N+3}\right|\ldots \right],$$

(i: i-th component of D_j_, N: the number bases D_j_)

a directionality for any D_j_ can be imposed;

$$D_{j}\left(5\to 3\right)=\left[C_{1}\left|G_{2}\right|A_{3}\left|C_{4}\right|\ldots \left|T_{i}\right|\ldots \left|A_{N-1}\right|T_{N}\left|E_{N+1}\right|E_{N+2}\left|E_{N+3}\right|\ldots \right],$$

and

$$D_{j}\left(3\to 5\right)=\left[T_{1}\left|A_{2}\right|\ldots \left|T_{N+1-i}\right|\ldots \left|C_{N-3}\right|A_{N-2}\left|G_{N-1}\right|C_{N}\left|E_{N+1}\right|E_{N+2}\left|E_{N+3}\right|\ldots \right].$$

The notation, ‘(5 → 3)’ and ‘(3 → 5)’, is simply an additional label representing the two possible types of endings of single-stranded DNA. Nonetheless, when the number of bases is finite, two sequences can be equivalent, as for example

$$D_{j}\left(5\to 3\right)=\left[C_{1}\left|G_{2}\right|A_{3}\left|C_{4}\right|T_{5}\left|A_{6}\right|T_{7}\right]$$

and

$$D_{j}\left(3\to 5\right)=\left[T_{1}\left|A_{2}\right|T_{3}\left|C_{4}\right|A_{5}\left|G_{6}\right|C_{7}\right],$$

unless the prime endings <5’(five prime) → 3’(three prime) > or <3’ → 5’ > accompanies the sequence designation.

In accordance with these postulates, we now can define the set D = {D_j_ (j = 1,2,3,…)| D_j_ ∈ C_5_ × C_5_ × C_5_ × … (N times, N ≤ ∞)} as the set of all possible sequences of recognized N-tuple single-stranded DNA bases. We can regard N to be a positive integer or infinity.

An analogous definition is clearly possible for the set R of RNA sequences; with ‘T’ substituted by ‘U’, operations of group Z_5_, are similarly definable because all results obtained for DNA pertain to RNA under the base substitution. Thus, set R = {R_j_ (j = 1,2,3,…)| R_j_ ∈ C_5_ × C_5_ × C_5_ × …(N times, N ≤ ∞)} is the set of all possible sequences of recognized N-tuple single-stranded RNA bases with C_5_ = {C, A, U, G, E}.

## §2 Group composition that yields changes in DNA bases via a Cartesian vector

Next, we can consider B = {B_m_ (m = 1,2,3,…) | B_m_ ∈ Z_5_× Z_5_× Z_5_ × …(an N-fold product, N = ∞)} = {Z_5_^×N^, ●}, where elements of B act on any D_j_. This means that D_j_ covers all possible sequences of the DNA bases, and this situation is the same for R_j_ of sequences of RNA bases.

Because B is a Cartesian product of the same Abelian group, it is also Abelian, where composition of any two elements of B is denoted by ‘●’ [4]. Details are shown in Appendix B and Figure 3. Accordingly, its formulation as a group B = {Z_5_^×N^, ●} is confirmed.

In a more general context, a Cartesian vector that is composed of the respective operators ‘b_(j→k)_’ that effects the change D_j_ into D_k_ is definable in the following way:

$$\begin{array}{l} B_{\left(j\to k\right)}=\left[b_{\left(j\to k\right)1}\left|b_{\left(j\to k\right)2}\right|b_{\left(j\to k\right)3}\left|\ldots \right|b_{\left(j\to k\right)i}\left|\ldots \right|b_{\left(j\to k\right)\left(N-1\right)}\left|b_{\left(j\to k\right)N}\right|n_{N+1}\left|n_{N+2}\right|n_{N+3}|\ldots \right],\\ \;\left(N:\mathrm{the}\;\mathrm{number}\;\mathrm{of}\;\mathrm{components}\right). \end{array}$$

Hence,

(4) $$‘D_{j}\circ B_{\left(j\to k\right)}=D_{k}’.$$

Clearly, for arbitrary ‘j’ and ‘k’, there exists a unique ‘m’ such that ‘B_(j→k)_ = B_m_ (m = 1,2,3,…)’; despite the difference in notation, the two are identical in practice.

Here, we present a simple example that consists of a multiple product of ‘B_(j→k)_’s. Consider the scenario that a certain sequence of a single strand (or one side of a double-strand) of DNA transitions from D_1_ to D_3_, in stepwise fashion,

$$\begin{array}{l} D_{1}=\left[A_{1}\left|C_{2}\right|C_{3}\left|G_{4}\right|T_{5}\left|E_{6}\right|E_{7}|\ldots \right]=\left[A_{1}\left|C_{2}\right|C_{3}\left(\;\right)G_{4}\left|T_{5}\right|E_{6}\left|E_{7}\right|\ldots \right],\\ D_{2}=[A_{1}\left|C_{2}\right|C_{3}\left(E_{4}|E_{5}\right)G_{6}\left|T_{7}\right|E_{8}\left|E_{9}\right|\ldots ],\\ D_{3}=[A_{1}\left|C_{2}\right|C_{3}\left(A_{4}|T_{5}\right)G_{6}\left|T_{7}\right|E_{8}\left|E_{9}\right|\ldots ],\\ D_{4}=[C_{1}\left|T_{2}\right|G_{3}\left(T_{4}|C_{5}\right)G_{6}\left|A_{7}\right|E_{8}\left|E_{9}\right|\ldots ]. \end{array}$$

We next consider the change ‘D_1_ → D_2_’. There exists an operator ‘B_(1→2)_ = [n_1_|n_2_|n_3_(**r**_**4**_|**u**_**5**_)l_6_|d_7_|**n**_**8**_|**n**_**9**_|…]’ that is able to produce this change, specifically, the insertion of two ‘E’s between ‘C_3_’ and ‘G_4_’ yields the change ‘D_1_ → D_2_’. However, this sort of manipulation can be troublesome. Hence, in our model, insertion/deletion of ‘E’s are instead ascribed to the way the vector D_j_ is interpreted. This is preferable as this avoids easier manipulations. Next, we construct the operator ‘B_(2→3)_’ that maps ‘D_2_ → D_3_’ (the details are shown in Appendix C). With reference to Figures 1, 2 and 3, we find

$$B_{\left(2\to 3\right)}=\left[n_{1}\left|n_{2}\right|n_{3}\left(u_{4}|d_{5}\right)n_{6}\left|n_{7}\right|n_{8}\left|n_{9}\right|\ldots \right].$$

In a similar manner,

$$B_{\left(3\to 4\right)}=\left[l_{1}\left|u_{2}\right|d_{3}\left(r_{4}|d_{5}\right)n_{6}\left|l_{7}\right|n_{8}\left|n_{9}\right|\ldots \right].$$

Naturally, the final D_4_ is obtained from D_1_ recursively,

(5) $$‘D_{1}\to D_{2}’,$$

(6) $$‘D_{2}\circ B_{\left(2\to 3\right)}●B_{\left(3\to 4\right)}=D_{4}’.$$

From the decomposition

(7) $$\begin{array}{l} ‘B_{\left(j\to k\right)}=B_{\left(j\to 0\right)}●B_{\left(0\to k\right)}={B_{j}}^{-1}●B_{k}’,\mathrm{we}\;\mathrm{obtain}‘D_{j}\circ B_{\left(j\to k\right)}=D_{j}\circ B_{\left(j\to 0\right)}●B_{\left(0\to k\right)}\\ \;=D_{0}●B_{\left(0\to k\right)}=D_{k}’, \end{array}$$

where

(8) $$D_{0}=\left[E_{1}\left|E_{2}\right|E_{3}\left|\ldots \right|E_{i}\left|\ldots \right|E_{N-1}\left|E_{N}\right|E_{N+1}\left|E_{N+2}\right|E_{N+3}|\ldots \right]$$

denotes the identity element of D.

**Figure 4:**
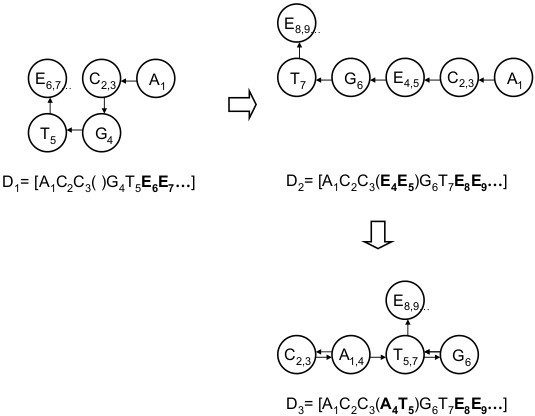
**Graphical representations for changes of DNA sequences.** Suppose next sequences; ‘D_1_ = [A_1_C_2_C_3_( )G_4_T_5_**E**_**6**_**E**_**7**_**E**_**8**_…]’, ‘D_2_ = [A_1_C_2_C_3_(**E**_**4**_**E**_**5**_)G_6_T_7_**E**_**8**_**E**_**9**_**E**_**10**_…]’ and ‘D_3_ = [A_1_C_2_C_3_(**A**_**4**_**T**_**5**_)G_6_T_7_**E**_**8**_**E**_**9**_**E**_**10**_…]’, a series of changes, ‘D_1_ → D_2_ → D_3_’ are drawn as three polygonal lines where each bases are linked also in the definition of group Z_5_. There, we recognize not the locations of ‘D’s but mere alphabets, indexed number and shapes.

Note that the group operations can act on D_j_ irrespective of whether the ‘E’s are explicit or implicit as defined in §1. Moreover, any sequence ‘D_j_’ can be presented as a polygonal line; as an example, the evolution of changes ‘D_1_ → D_3_’ is displayed in Figure 4.

## §3 Integration of a linear group and a rotational group as a wallpaper group

**Figure 5:**
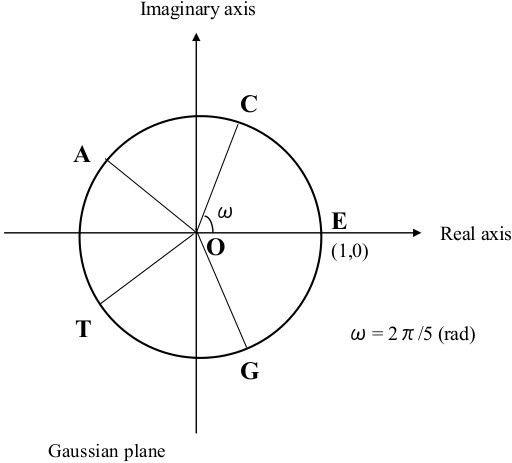
**A phasor diagram using the five bases over the Gaussian plane.** The five nucleic acid bases label the points equispaced on the unit circle to form the fivefold phasor diagram over the Gaussian plane. With ‘ω’ defining the counterclockwise rotation by ‘2π/5 (rad)’ around the origin in the Gaussian plane, composition of angles under modulo 5 addition generates a representation of the cyclic group. The complex units X = {Exp(m · ω · i)}, (i: imaginary unit, m: integer) following as a bijection angles to the plane; The bases are assigned to each phase: ‘Exp(0 · ω · i) = Exp(5 · ω · i) = 1 ↔ E’, ‘Exp(1 · ω · i) ↔ C’, ‘Exp(2 · ω · i) ↔ A’, ‘Exp(3 · ω · i) ↔ T’, ‘Exp(4 · ω · i) ↔ G’.

Looking at the definitions of groups Z_5_, D, and B, another approach is possible. The five bases can be represented by five equispaced phasors with a ‘2π/5’ angular phase separation located on the unit circle on the Gaussian plane, as depicted in Figure 5.

Herein, in the Gaussian plane, if ‘ω’ is defined to be the counterclockwise rotational angle ‘ω = 2π/5 (rad)’ and composition of ‘ω’ is denoted ‘●’, then assuming ‘ω’ obeys the ‘right translation rule’, we have

(9) $$\begin{array}{l} \omega =\omega_{1},\\ \omega \;●\;\omega =2\omega =\omega_{2},\\ \omega \;●\;\omega \;●\;\omega =3\omega =\omega_{3},\\ \omega \;●\;\omega \;●\;\omega \;●\;\omega =4\omega =\omega_{4},\\ \omega \;●\;\omega \;●\;\omega \;●\;\omega \;●\;\omega =5\omega =\omega_{5}=\omega_{0}=0\;\left(=\mathrm{no}\;\mathrm{rotation}\right). \end{array}$$

The general form of an arbitrary base is expressed as ‘X_m_ ↔ Exp(m · ω · i)’ (here, ‘i’ is the ‘imaginary unit’, ω = 2π/5 (rad), m = {0, 1, 2, 3, 4, 5}). With {*} meaning one of the bases among ‘C, A, T, G and E’, we construct the following map. Denoting composition by ‘◦’, ω_m_ acts on the identity trivially and hence yields the correspondences

(10) $$\begin{array}{l} \mathrm{Exp}\left(0\cdot i\right)↔\left\{\mathrm{Exp}\left(0\cdot \omega \cdot i\right)\right\}=\left\{1\right\}=\left\{1\right\}\circ \omega_{0}=E=X_{0},\\ \mathrm{Exp}\left(2\mathrm{\pi i}/5\right)↔\left\{\mathrm{Exp}\left(1\cdot \omega \cdot i\right)\right\}=\left\{1\right\}\circ \omega_{1}=C=X_{1},\\ \mathrm{Exp}\left(4\mathrm{\pi i}/5\right)↔\left\{\mathrm{Exp}\left(2\cdot \omega \cdot i\right)\right\}=\left\{1\right\}\circ \omega_{2}=A=X_{2},\\ \mathrm{Exp}\left(6\mathrm{\pi i}/5\right)↔\left\{\mathrm{Exp}\left(3\cdot \omega \cdot i\right)\right\}=\left\{1\right\}\circ \omega_{3}=T=X_{3},\\ \mathrm{Exp}\left(8\mathrm{\pi i}/5\right)↔\left\{\mathrm{Exp}\left(4\cdot \omega \cdot i\right)\right\}=\left\{1\right\}\circ \omega_{4}=G=X_{4},\\ X_{5}=X_{0}=E. \end{array}$$

Expanding the operations for ‘ω_1_, ω_2_, ω_3_, …’ on bases ‘C, A, T, G and E’, we establish for instance:

$$E\circ \omega_{1}=C,C\circ \omega_{1}=A,A\circ \omega_{2}=G,T\circ \omega_{3}=C,G\circ \omega_{1}=E.$$

In continuance, the set P_ω_ = {ω_1_, ω_2_, ω_3_, ω_4_, ω_0_ (= ω_5_)} is readily confirmed to form group {P_ω_, ●} where the identity element is ‘ω_0_’ and the inverse of ‘ω_m_’ is ‘ω_m_^−1^’:

(11) $$\begin{array}{l} {\omega_{0}}^{-1}=\omega_{0},\\ {\omega_{1}}^{-1}=\omega_{4},\\ {\omega_{2}}^{-1}=\omega_{3},\\ {\omega_{3}}^{-1}=\omega_{2},\\ {\omega_{4}}^{-1}=\omega_{1},\\ {\omega_{5}}^{-1}=\omega_{0}=0. \end{array}$$

**Figure 6:**
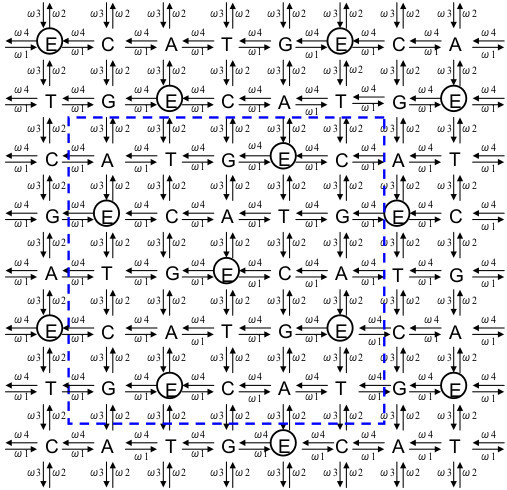
**Scheme for an accessorized wallpaper pattern synthesized from the linear group and rotational group for the five bases.** A bijection exists between the primitive operations of both groups: ‘r ↔ω = ω_1_, u ↔ 2ω = ω_2_, d ↔ 3ω = ω_3_, l ↔ 4ω = ω_4_, n ↔ ω_0_ = ω_5_ = 0’. Transitions in the four directions of the cruciform are expressible as rotations of the fivefold phasor diagram in the Gaussian plane. In other words, the linear operation and the rotational operation of the five bases are synthesized into a unique scheme (wallpaper pattern).

Closure and associativity follow from (9) and (10).Here, if we turn our attention to the wallpaper pattern, a further bijection obeying the postulates of the wallpaper group can be confirmed. Corresponding to Figures 3 and 6 a bijection between the Cayley Tables for translational and rotational operations can be established:

(12) $$\begin{array}{l} r\;↔\;\omega =\omega_{1},\\ u\;↔\;2\omega =\omega_{2},\\ d\;↔\;3\omega =\omega_{3},\\ l\;↔\;4\omega =\omega_{4},\\ n\;↔\;5\omega =\omega_{5}=\omega_{0}=0. \end{array}$$

Naturally, inverses (e.g., ω_2_^−1^ = ω_3_) are preserved in accordance with the inverses for ‘r, u, d, l, and n’. Any right translation of the horizontal line in Figures 1 and 6 (translational group) is also expressible as a rotation over the fivefold phasor diagram in Figure 5 (rotational group). Thus, these are able to be regarded as a synthesized form of the wallpaper style (wallpaper group) from which expressions such as ‘A◦r = A◦ω_1_ = T = E◦d’ and ‘A◦l = A◦ω_4_ = C = E◦r’ can be confirmed. All possible one-step changes between ‘A, C, T, G and E’ and ‘ω_1_, ω_2_, ω_3_, ω_4_ and ω_0_,’ are shown in Figure 2.

Therefore, this rule for ‘E’ does not break the postulates for set D, group Z_5_, and group B.

## §4 Methods to obtain complementary sequences from primary DNA

Suppose, from among ‘C, A, T, G and E’, a base ‘X_m_’ is given; its complementary base ‘X_m_^†^’ to ‘X_m_’ is defined as follows; for ‘X_m_ = {Exp(m · ω · i)}, m = {0, 1, 2, 3, 4, 5}, then ‘X_m_^†^’ is obtained by ‘X_m_^†^ = {Exp((5 – m) · ω · i)}’, where ‘{Exp(5 · ω · i)} = {1} = E’. In this regard,

(13) $$‘{X_{5}}^{†}=X_{0}={X_{0}}^{†}=X_{5}’$$

The procedure yields specifically ‘A^†^ = T’ and ‘C^†^ = G’.

Clearly, the complement of ‘E’ is ‘E’ itself; ‘E^†^ = E’.

Another notation for the ‘X_m_’ expressed as a base can be given. We introduce the one-value function ‘^ω^X(m)’ that provides the same results,

(14) $$‘X_{m}{=}^{\omega }X\left(m\right)=E\circ \mathrm{m\omega}=E\circ \left(\omega ●\omega ●\ldots ●\omega \;\left(m\;\mathrm{times}\right)\right)’\left(m=0,1,2,\ldots ,5\right).$$

As for ‘m’ in (14), both positive and negative integers are permissible. Thus,

‘X_m_^†^’ is expressible as

(15) $${X_{m}}^{†}{=}^{\omega }X\left(5–m\right)=E\circ \left(5–m\right)\omega =E\circ \left(\omega ●\omega ●\ldots ●\omega \;\left(‘5–m’\mathrm{times}\right)\right)’\left(m=0,1,2,3,4,5\right).$$

A simple example is illustrated below.

Suppose ‘D_j_’ = [A_1_|T_2_|C_3_|E_4_|G_5_|T_6_|…] = [^ω^X(2)|^ω^X(3)|^ω^X(1)|^ω^X(0)|^ω^X(4)|^ω^X(3)**|**…], then,

(16) $$\begin{array}{l} ‘{D_{j}}^{†}’=\left[{}^{\omega }X\left(5–2\right){\left|{}^{\omega }X\left(5–3\right)\right|}^{\omega }X\left(5–1\right){\left|{}^{\omega }X\left(5–0\right)\right|}^{\omega }X\left(5–4\right)|{}^{\omega }X\left(5–3\right)|\ldots \right],\\ \;=\left[{}^{\omega }X\left(3\right){\left|{}^{\omega }X\left(2\right)\right|}^{\omega }X\left(4\right){\left|{}^{\omega }X\left(0\right)\right|}^{\omega }X\left(1\right)|{}^{\omega }X\left(2\right)|\ldots \right],\\ \;=\left[T_{1}\left|A_{2}\right|G_{3}\left|E_{4}\right|C_{5}\left|A_{6}\right|\ldots \right]. \end{array}$$

In accordance with the wallpaper group in Figure 1, the translations in one direction (e.g., right) over a horizontal line form a cyclic group P_r_ that contains only {r, r^2^, r^3^, r^4^, r_e_ (= r^0^ = r^5^ = n)}. This group is isomorphic with group P_ω_ = {ω_1_, ω_2_, ω_3_, ω_4_, ω_0_ (= ω_5_)}, as is the group similarly generated over a vertical line.

Similar to ‘^ω^X(m)’, ‘X_m_’ can be expressed using another one-value function ^r^X(m) = E◦r^m^’:

(17) $$X_{m}{=}^{r}X\left(m\right)=E\circ r^{m}=E\circ \left(r●r●\ldots ●r\;\left(m\;\mathrm{times}\right)\right)\;\left(m=0,1,2,3,4,5\right).$$

Hence, ‘X_m_^†^’ (the complementary base of ‘X_m_’) is written as

(18) $${X_{m}}^{†}{=}^{r}X\left(5–m\right)=E\circ r^{5-m}=E\circ \left(r●r●\ldots ●r\;\left(‘5–m’\mathrm{times}\right)\right).$$

Extension to vertical translations is straightforward;

(19) $$‘X_{m}{=}^{u}X\left(5\right)=E\circ u^{5}=E\circ \left(u●u●\ldots ●u\;\left(m\;\mathrm{times}\right)\right),$$

and its complementary base ‘X_m_^†^’ can be identified similarly although the order of letters are somewhat different.

Consider the following simple example in identifying ‘D_j_^†^’ using ‘^r^X(m)’s;

$$\mathrm{for}\;‘D_{j}’=\left[A_{1}\left|T_{2}\right|C_{3}\left|E_{4}\right|G_{5}\left|T_{6}\right|\ldots \right]=\left[{}^{r}X\left(2\right){\left|{}^{r}X\left(3\right)\right|}^{r}X\left(1\right){\left|{}^{r}X\left(0\right)\right|}^{r}X\left(4\right)|{}^{r}X\left(3\right)|\ldots \right],$$

by replacing ‘^ω^X(m)’ by ‘^r^X(m)’ in formula (15), the same result is obtained.

According to these rules, ‘X_1_◦b_[X1→ X4]_ = X_1_◦r^3^ = E◦r^4^ = G’. In general, when the i-th component ‘b_[Xm1→ Xm2]i_’ of ‘B_(m1→m2)_’ changes X_m1_ (= E◦r^m1^ = E◦(m_1_ω)) to X_m2_ (= E◦r^m2^ = E◦(m_2_ω)).

Hence, the highlighted form of the operator vector is expressed as

(20) $$B_{\left(m1\to m2\right)}=\left[\ldots \left|b_{\left[\mathrm{Xm}1\to \mathrm{Xm}2\right]\;i}\right|\ldots \right]=\left[\ldots \left|{r^{m2–m1}}_{i}\right|\ldots \right]=\left[\ldots \left|\left(m_{2}–m_{1}\right)\omega_{\text{i}}\right|\ldots \right].$$

For a further example, given the operator ‘B_(j→k)_’ that changes D_j_ to D_k_,

$$\begin{array}{l} D_{j}=\left[C_{1}\left|A_{2}\right|E_{3}\left|\ldots \right|C_{i}\left|\ldots \right|T_{N-1}\left|A_{N}\right|E_{N+1}\left|E_{N+2}\right|E_{N+3}|\ldots \right],\\ \;=\left[E\circ {r^{1}}_{1}\left|E\circ {r^{2}}_{2}\right|E\circ {r^{0}}_{3}\left|\ldots \right|E\circ {r^{1}}_{i}\left|\ldots \right|E\circ {r^{3}}_{N-1}\left|E\circ {r^{2}}_{N}\right|E\circ {r^{0}}_{N+1}\left|E\circ {r^{0}}_{N+2}\right|E\circ {r^{0}}_{N+3}|\ldots \right],\\ D_{k}=\left[G_{1}\left|T_{2}\right|C_{3}\left|\ldots \right|G_{i}\left|\ldots \right|C_{N-1}\left|T_{N}\right|E_{N+1}\left|E_{N+2}\right|E_{N+3}|\ldots \right],\\ \;=\left[E\circ {r^{4}}_{1}\left|E\circ {r^{3}}_{2}\right|E\circ {r^{1}}_{3}\left|\ldots \right|E\circ {r^{4}}_{i}\left|\ldots \right|E\circ {r^{1}}_{N-1}\left|E\circ {r^{3}}_{N}\right|E\circ {r^{0}}_{N+1}\left|E\circ {r^{0}}_{N+2}\right|E\circ {r^{0}}_{N+3}|\ldots \right]. \end{array}$$

With details shown in Appendix D, ‘B_(j→k)_’ takes the form

$$B_{\left(j\to k\right)}=\left[{r^{3}}_{1}\left|{r^{1}}_{2}\right|{r^{1}}_{3}\left|\ldots \right|{r^{3}}_{i}\left|\ldots \right|{r^{3}}_{N-1}\left|{r^{1}}_{N}\right|{r^{0}}_{N+1}\left|{r^{0}}_{N+2}\right|{r^{0}}_{N+3}|\ldots \right].$$

Naturally, the state D_k_ is obtained through recursively applying the operations, D_j_ ◦B_(j→k)_ = D_k._ (Details are presented in Appendix D).

Whereas ‘D_j_^†^’s might have components in reverse order in terms of sense (5’ or 3’), there exists however certain ‘D_k_’ such that ‘D_k_ = D_j_^†^, (j, k = 0, 1, 2, 3, 4,…)’. With this, ‘D_j_^†^’ is one of the ordinal elements belonging to the same set D. Thus, the symbol ‘†’ need only be present when elements are distinct.

## §5 Further unifying notation to describe the wallpaper group operation

Consider Figure 6; we assume that the number of right translations ‘r’ ∈ group P_r_, (or ω ∈ group P_r_) is ‘a’ and the number of up translations ‘u’ ∈ group P (or 2ω (= ω_2_) ∈ group P_r_) is ‘b’ with a, b = …,-2, −1, 0, 1, 2,…. Similarly, with ‘d ↔ 3ω’ ‘l ↔ 4ω’, the total change can be summarized as ‘x[a, b]’. We can confirm that there exists at least a pair of ‘a, b’ that satisfies

(21) $$‘X=E\circ x\left[a,b\right]’,$$

because any base in Figure 6 can be obtained by a finite number of transitions from ‘E’. For instance, A can be expressed as; ‘A = E◦x[0, 1] = E◦x[2, 0] = E◦x[1,3] = E◦x[2,3] = E◦x[4,4]’. However, we remark that ‘x[a, b]’ means changes of bases from one to another prescribed by the wallpaper pattern. In practice, ‘x[a, b] = r^a^●u^b^’ constitutes a multiple composition of elements of group Z_5_. In addition, ‘x[−a, −b] = r^-a^●u^-b^ = l^a^●d^b^’ or ↔ ‘(−a)ω●(−b)(2ω) = (−a-2b)ω’. E.g., ‘x[−3, −2] means ‘r^−3^●u^−2^ = l^3^●d^2^’ or ↔ ‘(−3)ω●(−2 · 2)(ω) = (−3 -4)ω = (−7)ω = (−2)ω = 3ω = ω_3_’.

For the wallpaper group, the ‘a’ and ‘b’ should be interpreted in modulo 5 addition. The Cayley table for the wallpaper group are presented in Appendix A.

Within ‘the square unit cell’ in Figure 1 or 6, there are five pairs of ‘a, b’ for each base, as for ‘A’. Under modulo 5 addition, ‘x[a + 5, b + 5] = x[a, b]’ holds. Moreover, if ‘X^†^’ is obtained from ‘X’ using ‘X = E◦x[a, b]’, ‘X^†^’ can be determined as

(22) $$‘X^{†}=E\circ x^{-1}\left[a,b\right]=E\circ x\left[-a,-b\right]’,$$

(23) $$\mathrm{or}\;‘X^{†}=E\circ x\left[5-a,5-b\right]’,$$

because ‘X’ and ‘X^†^’ are symmetrically disposed with respect to ‘E’ over the wallpaper pattern that would be selected as a standard for the definition of ‘a, b’. In practice, for an arbitrary ‘X’, ‘X^†^’ can be obtained via (22) or (23) by making use of an arbitrary ‘E’ as the reference point for the symmetry.

For example, if ‘G = E◦x[1,2]’, then according to (22) ‘G^†^ = E◦x^−1^[1,2] = E◦x[−2, −1] = C or according to (23) G^†^ = E◦x[1,2] = E◦x[3,4] = C’. There are an infinite number of identifications for the complementary base for an arbitrary base ‘X’.

Moreover, if we define the composition for the ‘x[a, b]’s as

(24) $$‘x\left[a_{1},b_{1}\right]●x\left[a_{2},b_{2}\right]=x\left[a_{1},b_{1}\right]+x\left[a_{1},a_{2}\right]=x\left[a_{1}+a_{2},b_{1}+b_{2}\right]’,$$

we can confirm descriptions (20), (22) and (23). As for the operators ‘B_m_’, with ‘D_j_’ expressed as ‘D_j_ = […|E◦x[a_i_ , b_i_] _i_|…]’, one of the candidates of the appropriate ‘B_(j→j†)_’s that produces ‘D_j_◦B_(j→j†)_ = D_j_^†^’ is identified:

(25a) $$B_{\left(j\to j†\right)}=[\ldots |x[-2a_{i},-2b_{i}]{}_{i}|\ldots ]=\left[\ldots \left|r^{-2\mathrm{ai}}●{u^{-2\mathrm{bi}}}_{i}\right|\ldots \right]=\left[\ldots \left|l^{2\mathrm{ai}}●{d^{2\mathrm{bi}}}_{i}\right|\ldots \right],$$

(25b) $$\mathrm{or}=\left[\ldots \left|r^{-2\mathrm{ai}}●{{\left(r^{2}\right)}^{-2\mathrm{bi}}}_{i}\right|\ldots \right]=\left[\ldots \left|{r^{-2\mathrm{ai}-4\mathrm{bi}}}_{i}\right|\ldots \right]=\left[\ldots \left|{l^{2\mathrm{ai}+4\mathrm{bi}}}_{i}\right|\ldots \right]\;\left(\mathrm{using}\;‘u=r^{2}’,‘d=l^{2}’\right),$$

(25c) $$\mathrm{or}↔\left[\ldots |\left(-2a_{i}\right)\omega ●\left(-2b_{i}\right)\cdot {\left(2\omega \right)}_{\text{i}}\left|\ldots ]=[\ldots \right|\left(-2a_{i}-4b_{i}\right)\omega_{i}|\ldots \right]\;\left(\mathrm{using}‘r^{m}↔\mathrm{m\omega}’,‘u^{m}↔2\mathrm{m\omega}’\right).$$

The exponents ‘-2a_i_ -4b_i_’ in (25b) are permitted to take positive or negative integer values.

In these expressions, the rules for the wallpaper group (25a) can also be expressed as either for the linear group or for the rotational group (25b or 25c).

More generally,

‘D_j_ = […|E◦x[a_(j)i_ , b_(j)i_] _i_|…]’ is changed into ‘D_k_ = […|E◦x[a_(k)i_ , b_(k)i_] _i_|…]’, and

‘B_(j→k)_’s that provides ‘D_j_◦B_(j→k)_ = D_j_^†^’ is identified as

(26a) $$\begin{array}{l} B_{\left(j\to k\right)}=[\ldots |x[a_{\left(k\right)i}-a_{\left(j\right)i},b_{\left(k\right)i}-b_{\left(j\right)i}]{}_{i}|\ldots ]=\left[\ldots \left|r^{a\left(k\right)i-a\left(j\right)i}●{u^{b\left(k\right)i-b\left(j\right)i}}_{i}\right|\ldots \right]\\ \;\left(=\left[\ldots \left|l^{-a\left(k\right)i+a\left(j\right)i}●{d^{-b\left(k\right)i+b\left(j\right)i}}_{i}\right|\ldots \right]\right). \end{array}$$

(26b) $$\mathrm{Also},=\left[\ldots \left|r^{a\left(k\right)i-a\left(j\right)i}●{{\left(r^{2}\right)}^{b\left(k\right)i-b\left(j\right)i}}_{i}\right|\ldots \right]=\left[\ldots \left|r^{a\left(k\right)i-a\left(j\right)i}{{}^{+2b\left(k\right)i-2b\left(j\right)i}}_{i}\right|\ldots \right],$$

(26c) $$\mathrm{or}\;\mathrm{else}↔\left[\ldots \left|\left(a_{\left(k\right)i}-a_{\left(j\right)i}\right)\omega ●2\left(b_{\left(k\right)i}-b_{\left(j\right)i}\right)\omega_{i}\right|\ldots \right]=\left[\ldots \left|\left(a_{\left(k\right)i}-a_{\left(j\right)i}+2b_{\left(k\right)i}-2b_{\left(j\right)i}\right)\omega_{i}\right|\ldots \right].$$

As mentioned in §1, if a certain sequence ‘X’ has sense <5’ → 3’>, the complementary sequence ‘X^†^’ of a certain sequence ‘X’ is reversed to <3’ → 5’ > .

To aid understanding, we present the following examples: Given

$$\begin{array}{l} D_{j}=\left[A_{1}\left|T_{2}\right|E_{3}\left|\ldots \right|C_{i}\left|\ldots \right|G_{N-1}\left|A_{N}\right|E_{N+1}\left|E_{N+2}\right|\left|E_{N+3}\right|\ldots \right]\\ \;=\left[E\circ x{\left[0,\;1\right]}_{1}\left|E\circ x{\left[0,\;-1\right]}_{2}\right|\left|E\circ x{\left[0,\;0\right]}_{3}\right|\ldots \left|E\circ x{\left[1,\;0\right]}_{i}\right|\ldots \right)\\ \;\left(\ldots \left|E\circ x{\left[-1,\;0\right]}_{N-1}\right|E\circ x{\left[0,\;1\right]}_{N}\left|E\circ x{\left[0,\;0\right]}_{N+1}\right|E\circ x{\left[0,\;0\right]}_{N+2}\left|E\circ x{\left[0,\;0\right]}_{N+3}\right|\ldots \right]. \end{array}$$

then, according to (22), ‘D_j_^†^’ is simply

$$\begin{array}{l} {D_{j}}^{†}=[E\circ x^{-1}{\left[0,1\right]}_{1}|E\circ x^{-1}[0,-1]{}_{2}|E\circ x^{-1}[0,0]{}_{3}|\ldots \\ \;\ldots \left|E\circ x^{-1}{\left[1,0\right]}_{i}\right|\ldots |E\circ x^{-1}{\left[-1,0\right]}_{N-1}|E\circ x^{-1}[0,1]{}_{N}\left|E\circ x^{-1}{\left[0,0\right]}_{N+1}\right|E\circ x^{-1}{\left[0,0\right]}_{N+2}\left|E\circ x^{-1}{\left[0,0\right]}_{N+3}\right|\ldots ],\\ \;=[E\circ x[0,-1]{}_{1}|E\circ x{\left[0,1\right]}_{2}|E\circ x[0,0]{}_{3}|\ldots \\ \;\ldots |E\circ x{\left[-1,0\right]}_{i}\left|\ldots \right|E\circ x[1,0]{}_{N-1}|E\circ x[0,-1]{}_{N}\left|E\circ x{\left[0,0\right]}_{N+1}\right|E\circ x{\left[0,0\right]}_{N+2}\left|E\circ x{\left[0,0\right]}_{N+3}\right|\ldots ],\\ \;=\left[T_{1}\left|A_{2}\right|E_{3}\left|\ldots \right|G_{i}\left|\ldots \right|C_{N-1}\left|T_{N}\right|E_{N+1}\left|E_{N+2}\right|E_{N+3}|\ldots \right]. \end{array}$$

If we use the optional formula (25a − c), the relation ‘D_j_◦B_(j→j†)_ = D_j_^†^’ is derived. Details are given in Appendix E.Apart from these examples, additional identities for the wallpaper group can be verified using Figure 1 or 6; e.g.,

$$\begin{array}{l} ‘x\left[1,0\right]●x\left[0,1\right]=x\left[1,0\right]+x\left[0,1\right]\;\left(=r●u\right)=x\left[1+0,0+1\right]=x\left[1,1\right]=x[0,-1]\left(=d\right)’,\\ ‘x\left[2,0\right](=r●r)=x[0,1]=u’.‘x\left[3,1\right]●x\left[-1,10\right]=x[3-1,1+10]=x[2,11]=x[2,1]\\ =r^{2}●u^{1}=(r●r)●u=u●u=l’. \end{array}$$

We develop various general formulas:

(27) $$\begin{array}{l} ‘x\left[a+5,\;b\right]=x\left[a,\;b\right]’,\;‘x\left[a,\;b+5\right]=x\left[a,\;b\right]’,\\ ‘x\left[2a,\;-a\right]=x[0,\;0]’,\;‘x[a,\;2a]=x[0,0]’,\\ ‘x\left[-2a,\;a\right]=x[0,\;0]’,\;‘x[-a,\;-2a]=x[0,\;0]’. \end{array}$$

Other unknown rules might underlie the wallpaper pattern.

Concerning style in treating the wallpaper group, examples ‘X_m_ = ^r^X(m) in (16, 17, 19), and ^ω^X(m) in (14, 15, 20) could be regarded as a specific combination that are displayed as

(28) $$‘X_{m}{=}^{r}X\left(m\right)\;{=}^{\omega }X\left(m\right)=E\circ x\left[a,0\right]\;\left(a=\ldots ,-2,-1,0,1,2,\ldots ,5,6,\ldots ;\mathrm{integer}\right)’.$$

## §6 Treatment of changes of sequences and the insertion/deletion of DNA bases via an optionally generalized operation

Below, we demonstrate, using several examples containing ‘E’s, changes and inclusion/exclusion of DNA bases using a more generalized scheme.

For definiteness, let ‘D_j_’ be the sequence ‘**CGT**AT…C…TA’, we consider the change of its ‘1–3’ components ‘**C**_**1**_**G**_**2**_**T**_**3**_’ into ‘**G**_**1**_**T**_**2**_**A**_**3**_’, and moreover the insertion of two bases ‘GC’ between ‘T_3_’ and ‘A_4_’ denoted ‘( )’:

$$D_{j}=\left[C_{\left(j\right)1}\left|G_{\left(j\right)2}\right|T_{\left(j\right)3}\left(\;\right)A_{\left(j\right)4}\left|\ldots \right|C_{\left(j\right)i}\left|\ldots \right|T_{\left(j\right)N-1}\left|A_{\left(j\right)N}\right|E_{\left(j\right)N+1}\left|E_{\left(j\right)N+2}\right|E_{\left(j\right)N+3}|\ldots \right].$$

We denote the result of this transformation as D_k_,

(29) $$\begin{array}{l} D_{k}=[G_{\left(k\right)1}\left|T_{\left(k\right)2}\right|A_{\left(k\right)3}\left(G_{\left(k\right)4}|C_{\left(k\right)5}\right)A_{\left(k\right)6}\left|\ldots \right|C_{\left(k\right)i+2}\left|\ldots \right|T_{\left(k\right)N+1}\left|A_{\left(k\right)N+2}\right|E_{\left(k\right)N+3}\left|E_{\left(k\right)N+4}\right|\\ E_{\left(k\right)N+5}\ldots |\ldots ]. \end{array}$$

The procedure from D_j_ to D_k_ is described recursively to find operator ‘B_(j→h)_.

First, two ‘E’s are inserted after the 3rd component (this change is denoted ‘D_j_ → D_h_’) in preparation for insertion of ‘GC’;

(30) $$\begin{array}{l} D_{j}\to D_{h}=[C_{\left(h\right)1}\left|G_{\left(h\right)2}\right|T_{\left(h\right)3}\left(E_{\left(h\right)4}|E_{\left(h\right)5}\right)A_{\left(h\right)4+2}\left|\ldots \right|C_{\left(h\right)i+2}|\ldots \\ \;\ldots \left|T_{\left(h\right)N-1+2}\right|A_{\left(h\right)N+2}\left|E_{\left(h\right)N+1+2}\right|E_{\left(h\right)N+2+2}\left|E_{\left(h\right)N+3+2}\right|\ldots ]. \end{array}$$

Thus, ‘B_(j→h)_

$$=\left[b_{\left[C\to C\right]1}\left|b_{\left[G\to G\right]2}\right|b_{\left[T\to T\right]3}\left(b_{\left[G\to E\right]4}|b_{\left[C\to E\right]5}\right)b_{\left[A\to A\right]6}\left|\ldots \right|b_{\left[C\to C\right]i}\left|\ldots \right|b_{\left[T\to T\right]N-1}|b_{\left[A\to A\right]N}\right]’.$$

This change is in accordance with those rules for vector-like ‘D_j_’s dependent upon ‘E’s.

Hence, the operator B_(h→k)_ that produces the change from D_h_ to D_k_ is described as:

(31) $$\begin{array}{l} B_{\left(h\to k\right)}=[b_{\left[C\to G\right]1}\left|b_{\left[G\to T\right]2}\right|b_{\left[T\to A\right]3}\left(b_{\left[E\to G\right]4}|b_{\left[E\to C\right]5}\right)b_{\left[A\to A\right]6}|\ldots \\ \ldots \left|b_{\left[C\to C\right]i+2}\right|\ldots \left|b_{\left[T\to T\right]N+1}\right|b_{\left[A\to A\right]N+2}\left|b_{\left[E\to E\right]N+3}\right|b_{\left[E\to E\right]N+4}\left|b_{\left[E\to E\right]N+5}\right|\ldots ]. \end{array}$$

Thereby,

(32) $$\begin{array}{l} D_{h}\circ B_{\left(h\to k\right)}\\ =[C_{1}\circ b_{\left[C\to G\right]1}|G_{2}\circ b_{\left[G\to T\right]2}|T_{3}\circ b_{\left[T\to A\right]3}\left(E_{4}\circ b_{\left[E\to G\right]4}|E_{5}\circ b_{\left[E\to C\right]5}\right)A_{6}\circ b_{\left[A\to A\right]6}|\ldots |C_{i}\circ b_{\left[C\to C\right]i+2}|\ldots \\ \;|T_{N+1}\circ b_{\left[T\to T\right]N+1}|A_{N+2}\circ b_{\left[A\to A\right]N+2}|E_{N+3}\circ b_{\left[E\to E\right]N+3}|E_{N+4}\circ b_{\left[E\to E\right]N+4}|E_{N+5}\circ b_{\left[E\to E\right]N+5}|\ldots ]. \end{array}$$

With reference to Figure 1, 2, 6 or Appendix A,

(33) $$\begin{array}{l} =[C_{1}\circ d_{1}\left|G_{2}\circ l_{2}\right|T_{3}\circ l_{3}\left(E_{4}\circ l_{4}|E_{5}\circ r_{5}\right)A_{6}\circ n_{6}\left|\ldots \right|C_{i+2}\circ n_{i+2}|\ldots \\ \;\ldots \left|T_{N+1}\circ n_{N+1}\right|A_{N+2}\circ n_{N+2}\left|E_{N+3}\circ n_{N+3}\right|E_{N+4}\circ n_{N+4}\left|E_{N+5}\circ n_{N+5}\right|\ldots ], \end{array}$$

(34) $$\begin{array}{l} =\left[G_{1}\left|T_{2}\right|A_{3}\left(G_{4}|C_{5}\right)A_{6}\left|\ldots \right|C_{i+2}\left|\ldots \right|T_{N+1}\left|A_{N+2}\right|E_{N+3}\left|E_{N+4}\right|E_{N+5}|\ldots \right],\\ =D_{k}.\text{(29)}. \end{array}$$

This indicates a code change of the ‘1–3’ components and a ‘GC’ insertion after the 3rd as described via the two steps: 1) D_j_ → D_h_ (inserting two ‘E’s after the ‘3rd’ component), and 2) D_h_ ◦B_(h→k)_ = D_k_. Note that the exclusion of the ‘4–5’ components ‘GC’ from D_k_ and the transformation of the ‘1–3’ components from ‘GTA’ to ‘CGT’ constitute the recursive procedure for the inverse operator

(35) $$‘B_{\left(k\to h\right)}={B_{\left(h\to k\right)}}^{-1}’$$

Alternatively, ‘D_h_ → D_j_’ is obtained by deleting the two ‘E’s from the ‘4–5’ components of D_h_ to yield the initial state ‘D_j_’ in accordance with the characteristics of the vector-like ‘D_j_’s.

In summary, essentially, all transitions (changes and inclusion/exclusion) of a certain sequence within the same single-stranded DNA, whether it has finite or infinite length, can be described in principle within a single operation using only the unique operator B_(… →… )_ ∈ group B.

## §7 Synthesis of changes, insertion/deletion, and recombination of DNA bases

As a further development, to demonstrate recombination, take two finite sequences ‘GETAGT (= D_c1_)’ and ‘ATAGCTA (= D_d1_)’. These have vector expressions

(36) $$\begin{array}{l} D_{c1}=\left[G_{1}E_{2}\underline{T_{3}A_{4}G_{5}T_{6}}|E_{7}E_{8}E_{9}\ldots \right],\\ \;=\left[G_{\left(c1\right)1}\left|E_{\left(c1\right)2}\right|T_{\left(c1\right)3}\left|A_{\left(c1\right)4}\right|G_{\left(c1\right)5}\left|T_{\left(c1\right)6}\right|E_{\left(c1\right)7}\left|E_{\left(c1\right)8}\right|E_{\left(c1\right)9}|\ldots \right], \end{array}$$

(37) $$\begin{array}{l} D_{d1}=\left[A_{1}T_{2}\underline{A_{3}G_{4}C_{5}T_{6}A_{7}}|E_{8}E_{9}E_{10}\ldots \right],\\ \;=\left[A_{\left(d1\right)1}\left|T_{\left(d1\right)2}\right|A_{\left(d1\right)3}\left|G_{\left(d1\right)4}\right|C_{\left(d1\right)5}\left|T_{\left(d1\right)6}\right|A_{\left(d1\right)7}\left|E_{\left(d1\right)8}\right|E_{\left(d1\right)9}\left|E_{\left(d1\right)10}\right|\ldots \right]. \end{array}$$

To illustrate for the pair D_c1_ and D_d1_, we consider recombination to take place between the sequence ‘T_3_A_4_G_5_T_6_’ of the (3–6)-th component of ‘D_c1_’ and the ‘AGCTA’ of the (3–7)-th component of ‘D_d1_’ at the same instant.

First, in the pair of sequences, a series of ‘E’s of complementary size is inserted in ‘D_c1_’ just before the sequence to be converted, and in ‘D_d1_’ just after the sequence to be converted. For example, for ‘D_c1_’, five ‘E’s, ‘**EEEEE**’, of size equivalent to that of ‘**A**_**3**_**G**_**4**_**C**_**5**_**T**_**6**_**A**_**7**_’ of ‘D_d1_’, are inserted just before ‘T_3_’ in ‘D_c1_’ where ‘**A**_**3**_**G**_**4**_**C**_**5**_**T**_**6**_**A**_**7**_’ is to be located, that is, the interval between ‘the 2nd ‘E_2_’ and 3rd ‘T_3_’ within ‘D_c1_’. Under this procedure, D_c1_ changes into D_c2_:

(38) $$\begin{array}{l} D_{c2}=[G_{1}E_{2}\left(\mathrm{EEEEE}\right)\underline{T_{3+5}A_{4+5}G_{5+5}T_{6+5}}|E_{7+5}E_{8+5}E_{9+5}\ldots ],\\ \;=[G_{1}E_{2}\left(\mathrm{EEEEE}\right)\underline{T_{8}A_{9}G_{10}T_{11}}|E_{12}E_{13}E_{14}\ldots ]. \end{array}$$

Here, we assume that ‘**EEEEE**’ is changed into ‘**A**_**3**_**G**_**4**_**C**_**5**_**T**_**6**_**A**_**7**_’ (originally, the (3–7)-th component of ‘D_d1_’). In addition, ‘T_8_A_9_G_10_T_11_’ is transformed into the same number of ‘E’s, ‘EEEE’, at the same time. By this process, ‘D_c2_’ changes in ‘D_c3_’:

(39) $$D_{c3}=\left[G_{1}E_{2}\left(A_{3}G_{4}C_{5}T_{6}A_{7}\right)\underline{E_{8}E_{9}E_{10}E_{11}}|E_{12}E_{13}E_{14}\ldots \right].$$

Note that bold type and underline are here merely pedagogical aids to help identify sequence changes. Meanwhile, four ‘E’s ‘**EEEE**’ equivalent in size to ‘T_3_A_4_G_5_T_6_’ of ‘D_c1_’ would be inserted after ‘A_7_’ of ‘D_d1_’ where ‘T_3_A_4_G_5_T_6_’ of ‘D_c1_’ is to be located within ‘D_d1_’. That is, ‘T_3_A_4_G_5_T_6_’ is inserted into the interval between the 7th ‘A_7_’ and 8th ‘E_8_’ within ‘D_d1_’. In this procedure, D_d1_ changes into D_d2_:

(40) $$\begin{array}{l} D_{d2}=[A_{1}T_{2}\underline{A_{3}G_{4}C_{5}T_{6}A_{7}}\left(\mathrm{EEEE}\right)E_{8+4}E_{9+4}E_{10+4}\ldots ],\\ \;=[A_{1}T_{2}\underline{A_{3}G_{4}C_{5}T_{6}A_{7}}\left(\mathrm{EEEE}\right)E_{12}E_{13}E_{14}\ldots ]. \end{array}$$

Furthermore, we change ‘EEEE’ into the equivalent-sized ‘**T**_**8**_**A**_**9**_**G**_**10**_**T**_**11**_’ (originally, the (3-6)-th components of ‘D_c1_’) while ‘A_3_G_4_C_5_T_6_A_7_’ is transformed into the equivalent-sized ‘EEEEE’. Through this procedure, ‘D_d2_’ changes in ‘D_d3_’:

(41) $$D_{d3}=\left[\underline{A_{1}T_{2}E_{3}E_{4}E_{5}E_{6}E_{7}}\left(T_{8}A_{9}G_{10}T_{11}\right)E_{12}E_{13}E_{14}\ldots \right]$$

As a result, if we omit the infinite series of ‘E’s from right end, we have the recombination (partial conversion between this pair of sequences from ‘D_c1_, D_d1_’) with ‘D_c1_’ = ‘G_1_E_2_T_3_A_4_G_5_T_6_’ being transformed into ‘D_c3_’ = ‘G_1_E_2_A_3_G_4_C_5_T_6_A_7_’ and ‘D_d1_’ = ‘A_1_T_2_A_3_G_4_C_5_T_6_A_7_’ being transformed into ‘D_d3_’ = ‘A_1_T_2_T_3_A_4_G_5_T_6_’. We define the manipulation of the recombination (partial/total conversion) between ‘D_c1_, D_d1_’ in this way.

In the initial stage in the previous illustration, we inserted different sizes of ‘E’ sequences in each line; however, processes ‘D_c1_ → D_c2_’ and ‘D_d1_ → D_d2_’ are preferred to be regarded as ‘E’ insertions/deletions (see comments prior to equation (5)) and this rule depends upon the characteristics of these vectors (e.g., ‘D_j_’s).

As previously explained, the operations can be performed in any of the three equivalent linear group, rotational group, and wallpaper group. Choosing the wallpaper group,

(42) $$\begin{array}{l} D_{c2}\circ B_{\left(c2\to c3\right)}\\ =[G_{1}\circ n_{1}|E_{2}\circ n_{2}\left(E_{3}\circ u_{3}\left|E_{4}\circ l_{4}\right|E_{5}\circ r_{5}\left|E_{6}\circ d_{6}\right|E_{7}\circ u_{7}\right)\underline{T_{8}\circ u_{8}}|\underline{A_{9}\circ d_{9}}|\underline{G_{10}\circ r_{10}}|\underline{T_{11}\circ u_{11}}\left|E_{12}\circ n_{12}\right|E_{13}\circ n_{13}\left|E_{14}\circ n_{14}\right|\ldots ],\\ =[G_{1}|E_{2}\left(A_{3}\left|G_{4}\right|C_{5}\left|T_{6}\right|A_{7}\right)\underline{E_{8}}|\underline{E_{9}}|\underline{E_{10}}|\underline{E_{11}}\left|E_{12}\right|E_{13}\left|E_{14}\right|\ldots ], \end{array}$$

(43) $$\begin{array}{l} =D_{c3},\\ \mathrm{where}\;B_{\left(c2\to c3\right)}=[n_{1}|n_{2}\left(u_{3}\left|l_{4}\right|r_{5}\left|d_{6}\right|u_{7}\right)\underline{u_{8}}|\underline{d_{9}}|\underline{r_{10}}|\underline{u_{11}}\left|n_{12}\right|n_{13}\left|n_{14}\right|\ldots ]. \end{array}$$

Also,

(44) $$\begin{array}{l} D_{d2}\circ B_{\left(d2\to d3\right)}\\ =[A_{1}\circ n_{1}\left|T_{2}\circ n_{2}\right|\underline{A_{3}\circ d_{3}}|\underline{G_{4}\circ r_{4}}|\underline{C_{5}\circ l_{5}}|\underline{T_{6}\circ u_{6}}|\underline{A_{7}\circ d_{7}}\\ \;\left(E_{8}\circ d_{8}\left|E_{9}\circ u_{9}\right|E_{10}\circ l_{10}|E_{11}\circ d_{11}\right)E_{12}\circ n_{12}\left|E_{13}\circ n_{13}\right|E_{14}\circ n_{4}|\ldots ],\\ =[A_{1}\left|T_{2}\right|\underline{E_{3}}|\underline{E_{4}}|\underline{E_{5}}|\underline{E_{6}}|\underline{E_{7}}\left(T_{8}\left|A_{9}\right|G_{10}|T_{11}\right)E_{12}\left|E_{13}\right|E_{14}|\ldots ], \end{array}$$

(45) $$\begin{array}{l} =D_{d3},\\ \mathrm{where}\;B_{\left(d2\to d3\right)}=[n_{1}\left|n_{2}\right|\underline{d_{3}}|\underline{r_{4}}|\underline{l_{5}}|\underline{u_{6}}|\underline{d_{7}}\left(d_{8}\left|u_{9}\right|l_{10}|d_{11}\right)n_{12}\left|n_{13}\right|n_{14}|\ldots ]. \end{array}$$

With respect to (42) and (44), the inverse identities are confirmed:

(46) $${B_{\left(c2\to c3\right)}}^{-1}=B_{\left(d2\to d3\right)}.$$

Generally, B_(_→_)_ giving transition ‘D_c2_ → D_c3_’ automatically produces an inverse change for ‘D_d2_ → D_d3_’, as stated in (46) and reduces troublesome manipulations, even if only partially.

## §8 Further applications of the composition category-like prototypal model using additional ribonucleic acid (RNA)

We next comment on other possible applications of the model. The category theory-like construction for treating DNA transcription to RNA might be conceivable, and the combination of the set and the group can comprise a category when these satisfy category theory postulates [48,49]. That is because we believe that in future developments the discussion should embrace category theory as one of the important options.

To begin, according to our description for handling ‘E’s, it seems difficult to define inverse elements in a group theoretical way when there are deletions of ‘E’s from any place in a sequence because we cannot find sufficient numbers of ‘E’s in the target component of D_j_.

**Figure 7:**
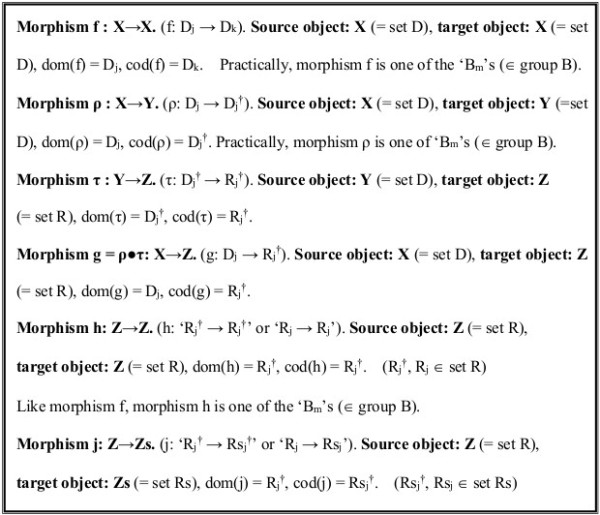
**Definition of category C.** A simplistic definition of category C treating the traditional “central dogma” is presented. In practice, any selection of operations (morphisms) is permissible because composition within the category C using any of the three operations belonging to the ‘linear group’, ‘rotational group’ or ‘wallpaper group’ is considered possible. The morphisms f and h correspond to elements ‘B_m_’ of group B, the only difference being the ‘T’s and ‘U’s. Actually, morphism j is regarded as part of morphism h (‘R_j_^†^’s and ‘set R’ are also substitutable for ‘Rs_j_^†^’s and ‘set Rs’); all morphisms except for ‘τ’ an ‘g’ satisfy the group postulates, and are treated as operations of group B.

Thus, we consider the ‘morphism f’ that transforms the sequence of DNA bases within set D as follows [48,49].

morphism f : X → X, dom(f) = D_j_, cod(f) = D_k_. Object ‘X’ is the set of ‘D_j_’s. There exists a morphism ‘1_X_’ such that ‘1_X_●f = f = f●1_X_’ for every ‘morphism f’, when ‘1_X_’ = [n_1_|n_2_|n_3_|…|n_i_|…|n_N-1_|n_N_|…] (∈ group B). If supplemented, the ‘morphisms f’ comprise ‘group B’ (see reference list in Figure 7). The group composition for ‘f_1_’ and ‘f_2_’ is denoted ‘f_1_●f_2_’.

**Figure 8:**
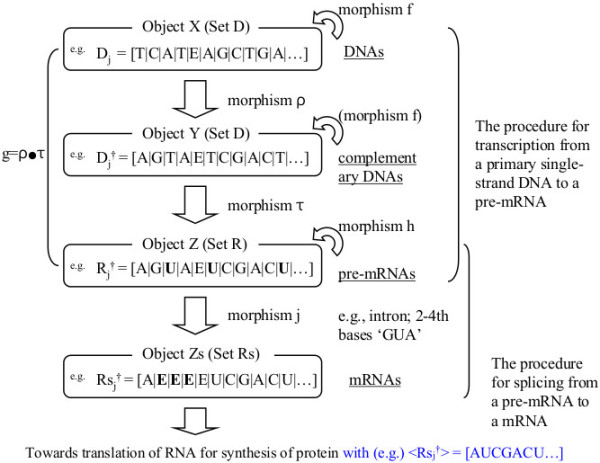
**Example of a category theory-like scheme on canonical “central dogma” and reference chart.** The features of DNA sequencing involving changes, insertion/deletion, and recombination, can receive a group theoretic treatment. A single-stranded ‘D_j_’ (“sense” of double-stranded DNA) is transformed into its complementary sequence ‘D_j_^†^’ (same as its ‘anti-sense’) or remains in the same ‘D_j_’ by morphism ρ. A single-stranded ‘D_j_^†^’ (same as the “anti-sense” of ‘D_j_’) without discrimination for directions (<5’ → 3’ > or <3’ → 5’>) is transformed into its pre-messenger RNA (pre-mRNA; ‘R_j_^†^’) containing ‘introns’ that are not used in protein synthesis, or remains in the same ‘D_j_^†^’ by morphism τ. Then, ‘D_j_^†^’ is transcribed into mature RNA (mRNA; ‘Rs_j_^†^’) through RNA splicing. After either the simultaneous deletion of all explicit ‘E’s from ‘Rs_j_^†^’s, <Rs_j_^†^ > _1_ (= < Rs_j_^†^>), or through a sequence of deletions, <Rs_j_^†^ > _t_ (t = 0, 1, 2,…) with additional idels, protein synthesis can be described in subsequent procedures of this scheme. Objects X and Y are in set D, and object Z is in set R; however, object Zs is in set Rs, which is a part of set Z. Over set Rs, group operations are not definable at present.

As mentioned earlier, sequences of DNA consisting of bases ‘C, A, T, and G’ are transcribed into RNA consisting of ‘C, A, U, and G’. This process can be regarded as the combination of two manipulation; Ι) transcription from the original DNA sequences (D_j_) to those of its complement (D_j_^†^), and ΙΙ) alternation from ‘C, A, T, and G’ to ‘C, A, U, and G’ (D_j_^†^ → R_j_^†^) (both are illustrated in Figure 8).

### Step Ι

The transformation from the original DNA bases ‘D_j_’ into the complementary sequence ‘D_j_^†^’ (e.g., ‘TCATEAGCTGA…’ → ‘AGTAETCGACT…’) (for transcription to pre-messenger RNA (pre-mRNA) before splicing) can be performed via the manipulation (13–15, 17, 18, 20, 22, 23, 25a–c) in §3 and §4. ‘D_j_^†^’ can be obtained via the linear group (17, 18, 20), the rotational group (14, 15, 20) and also the wallpaper group (21–23, 25a–c, 26a–c). Thereby, morphism ρ : X → Y, dom(ρ) = D_j_, cod(ρ) = D_j_^†^. Object ‘Y’ is the set of ‘D_j_^†^’s (essentially equivalent to the set of ‘D_j_’s).

There exist morphisms ‘1_X_’ and ‘1_Y_’ such that ‘1_Y_●ρ = ρ = ρ●1_X_’ for ‘morphism ρ’, where

(47) $$‘1_{X}=1_{Y}=\left[n_{1}\left|n_{2}\right|n_{3}\left|\ldots \right|n_{i}\left|\ldots \right|n_{N-1}\left|n_{N}\right|\ldots \right]’.$$

However, in practice, morphism ρ is one of the ‘B_m_’s ∈ group B (see Figures 7 and 8).

### Step ΙΙ

Next, we define manipulations that change the above ‘D_j_^†^’ into ‘R_j_^†^’ where all ‘T’s are converted into ‘U’s; e.g., (D_j_^†^=) [A|G|**T**|A|E|**T**|C|G|A|C|**T**|…] → (R_j_^†^=) [A|G|**U**|A|E|**U**|C|G|A|C|**U**|…]’.

This process can also be expressed in a similar way as transcription.

morphism τ : Y → Z, dom(τ) = D_j_^†^, cod(ρ) = R_j_^†^. Object ‘Z’ is the set of ‘R_j_^†^’s. There exist morphisms ‘1_Y_’ and ‘1_Z_’ such that ‘1_Z_●τ = τ = τ●1_Y_’ for every ‘morphism τ’, where

(48) $$‘1_{Y}=1_{Z}=\left[n_{1}\left|n_{2}\right|n_{3}\left|\ldots \right|n_{i}\left|\ldots \right|n_{N-1}\left|n_{N}\right|\ldots \right]’.$$

(refer to Figures 7 and 8).

Evidently, morphism τ does not satisfy the group postulates because the source object ‘Y’ and target object ‘Z’ are different and a single set of operations cannot be defined at this stage.

Additionally, as for Steps І and ΙΙ, the resultant process for morphisms ρ and τ can be expressed as:

(49) morphismg=ρ●τ:X→Z,

dom(g) = D_j_, cod(g) = R_j_^†^ (see Figures 7 and 8).

(50) $$\mathrm{There}\;\mathrm{exist}\;\mathrm{morphisms}\;‘1_{X}’\;\mathrm{and}\;‘1_{Z}’\;\mathrm{such}\;\mathrm{that}\;‘1_{Z}●g=g=g●1_{X}’.$$

The only difference between D_j_^†^ and R_j_ is the appearance ‘T’ and ‘U’ in the sequences.

Naturally, for RNA base sequences, similar treatments are possible in the single group B:

morphism h : Z → Z, dom(h) = R_j_^†^, cod(h) = R_k_^†^ (Figures 7 and 8).

(51) $$\mathrm{There}\;\mathrm{exists}\;\mathrm{morphism}\;‘1_{Z}’\;\mathrm{such}\;\mathrm{that}\;‘1_{Z}●h=h=h●1_{Z}’.$$

Ordinarily, in prokaryotic cells, the DNA sequences are transcribed along their entire length. For eukaryotic cell, a splicing process is needed using nascent pre-messenger RNA (pre-mRNA) where introns of DNA bases are removed and exons are joined before producing a correct protein through translation, resulting in the mature messenger RNA (mRNA). Thus, the previous procedure was about the prokaryotic cell or the pre-translation of pre-mRNA in the eukaryotic cell. Therefore, to treat the products after this RNA splicing procedure in the eukaryotic cell, the following approach might be possible. The removal of introns can be regarded as changes from a certain series of bases to ‘E’s as follows.

If ‘**GUA**’ is removed from ‘A(**GUA**)EUCGACU…’ to become **‘**A( )EUCGACU…’, this procedure can be described as; ‘R_j_^†^ → Rs_j_^†^’,

(52) $$‘{R_{j}}^{†}=\left[A_{1}\left(G_{2}\left|U_{3}\right|A_{4}\right)E_{5}\left|U_{6}\right|C_{7}|G_{8}\left|A_{9}\right|C_{10}\left|U_{11}\right|E_{12}\left|E_{13}\right|E_{14}|\ldots \right]’,$$

(53) $$‘R{s_{j}}^{†}=\left[A_{1}\left(E_{2}\left|E_{3}\right|E_{4}\right)E_{5}\left|U_{6}\right|C_{7}|G_{8}\left|A_{9}\right|C_{10}\left|U_{11}\right|E_{12}\left|E_{13}\right|E_{14}|\ldots \right]’.$$

The ‘Rs_j_^†^’ form a set Rs = {Rs_j_^†^ (j = 1,2,3,…)} that is a part of set R (see Figures 7 and 8).

Hereon, we admit ‘E’s in the sequences of RNAs (as elements of set Rs) during the operations before morphism ‘f’ and after morphism ‘j’ to maintain theoretical consistency. Thus, if the result of a series of these maps is ‘Rs_j_^†^ = A_1_**E**_**2**_**E**_**3**_**E**_**4**_E_5_U_6_C_7_G_8_A_9_C_10_U_11_**E**_**12**_**E**_**13**_**E**_**14**_…’, then the actual RNA sequence should be interpreted as ‘AUC…’. Specifically, an equivalent-sized substitution of some bases in pre-mRNA with ‘E’s can be written morphism j: Z → Zs, dom(j) = R_j_^†^, cod(j) = Rs_j_^†^. There exists a morphism ‘1_Zs_’ such that ‘1_Zs_●j = j = j●1_Zs_’.

‘j’ changes some series of bases from ‘C, A, U, G, E’ to an equivalent-sized series of ‘E’s within the partial operations of the group B. However, morphism ‘j’ fails the group axioms, as inverse might not be definable.

Finally, as in §1, we apply the simultaneous deletions of all explicit ‘E’s of mRNA other than the trailing ‘E’s, the state after these deletions being denoted with ‘< >’; for

$$\begin{array}{l} ‘R{s_{j}}^{†}=\left[A_{1}E_{2}E_{3}E_{4}E_{5}U_{6}C_{7}G_{8}A_{9}C_{10}U_{11}E_{12}E_{13}E_{14}\ldots \right]\\ \;=[A_{1}\left(E_{2}E_{3}E_{4}\right)E_{5}U_{6}C_{7}G_{8}A_{9}C_{10}U_{11}E_{12}E_{13}E_{14}\ldots ]’, \end{array}$$

the description ‘<Rs_j_^†^ > = [A_1_U_2_C_3_G_4_A_5_C_6_U_7_…]’ is specified without explicit non-trailing ‘E’s. In this regard, as in §1, if some indels (insertions/deletions) occur at certain bases of < Rs_j_^†^>, as for ‘<Rs_j1_^†^ > = [A_1_U_2_(**E**_**3**_)G_4_(**E**_**5**_)C_6_U_7_…]’ (with the deletion of ‘C_3_’ and ‘A_5_’), we state the result as ‘<<Rs_j1_^†^>> = [A_1_U_2_G_3_C_4_U_5_…]’. ‘R_j_^†^’s include < Rs_j_^†^ > s and ‘<<Rs_j_^†^> > s from the set R and both still satisfy the postulates of group B. This rule is a relative postulate and explicit ‘E’s are not absolutely forbidden in ‘<Rs_j_^†^>’s or ‘<<Rs_j_^†^>>’s, hence further indels of ‘E’s into ‘<Rs_j_^†^>’s or ‘<<Rs_j_^†^>>’s are not forbidden.

Also, omissions of explicit ‘E’s are considered as in ‘{<Rs_j1_^†^>} = [A_1_U_2_G_4_C_6_U_7_…], where place numbers ‘3’ and ‘5’ are absent indicating implicitly their presence in the vector. (Note that all products belong to group B.) Similarly, t-tuples of ‘< >’s are denoted ‘<<<<Rs_j_^†^>>>> (t-tuple) = <Rs_j_^†^> _t_’ representing multiple deletions of ‘E’s (t-times). Combinations of symbols ‘{ }’ and ‘< >’ are also allowed when necessary, as for example {<{{<Rs_j1_>}}>}, as long as the subscripted place numbers are adequately recognized/traced.

Nevertheless, the multiple use of ‘< >’ to remove all ‘E’s in the vector ‘Rs_j_^†^’ should have a unique meaning with regard to protein synthesis. As a result, the subsequent reading/translation in line with ‘codon’-like ‘[AUC|GAC|U…]’, ‘[A|UCG|ACU|…]’ or ‘[AU|CGA|CU…]’ leads in an ordinal way to a description of protein synthesis. Through the use of ‘{ }’ and/or ‘< >’, the concept ‘E’ may have benefits, although this may need to be intensely explored in future studies.

The procedure reversing transcription, found for example in retrovirus, is also describable if additional options are added to the scheme. However, these options are omitted at this stage to keep the model simple.In summary, suppose we have a ‘category C’ with objects ‘X’, ‘Y’, ‘Z’, ‘Zs’ and morphisms ‘f’ , ‘ρ’ , ‘τ’ , ‘g’ , ‘h’ and ‘j’. We affirm that these definitions satisfy the postulates of category. A list is given in Figure 7. Indeed, morphisms other than ‘τ’ and ‘g’ are simple group-theoretical products. One of the reasons we have introduced the concept ‘category’ is that the translation from single-strand DNAs to RNAs is difficult or impossible to systematize as a group structure. Therefore, if we identify the differences, we can treat all manipulations, except for ‘τ’ and ‘g’, based simply on group B.

The expression ‘hom(X, X)’ denotes all morphisms f: ‘from X to X’. Likewise, ‘hom(X, Y)’ denotes all morphisms ρ: ‘from X to Y’. In addition, ‘hom(Y, Z)’ denotes all morphisms τ: ‘from Y to Z, and hom(X, Z) denotes all morphism g: from X to Z. Then, hom(Z, Z) denotes all morphisms h: ‘from Z to Z’. Finally, hom(Y, Z) denotes all morphism h: from Y to Z. (Details are displayed in Appendix F)

As is explained in §3 and §4, the rotational group can be regarded as a specific bijection of the wallpaper group [2,44-47], so, we can describe this relationship naturally in a category theory-like way where two categories C_1_ and C_2_ are linked.

First, we consider two categories C_1_ and C_2_ with a ‘functor F’ from C_1_ to C_2_ written ‘F: C_1_ → C_2_’. For example, the pre-category C is denoted C_1_ and the product of functor F on category C_1_ is denoted C_2_[48,49]. Note that the only difference between C_1_ and C_2_ is assumed to be the nature of its expression; morphism f_1_ = B_1_ (∈ category C_1_) is based on the wallpaper group in Figure 1 or 6; e.g.,

(54) $$‘B_{1}=\left[r_{1}\left|l_{2}\right|u_{3}\left|\ldots \right|n_{i}\left|\ldots \right|r_{N-1}\left|d_{N}\right|\ldots \right]=\left[\ldots \left|x{\left[a_{i},\;b_{i}\right]}_{i}\ldots \right|\right]=\left[\ldots \left|r^{\mathrm{ai}}●{u^{\mathrm{bi}}}_{i}\right|\ldots \right]\;\left(\in \;\mathrm{group}\;B_{1}\right)’.$$

Additionally, morphism f_2_ = B_2_ (∈ category C_2_) is based on the rotational group over the Gaussian plane in Figure 5; e.g.,

(55) $$‘B_{2}=\left[\omega_{1\;1}\left|\omega_{4\;2}\right|\omega_{2\;3}\left|\ldots \right|\omega_{0\;i}\left|\ldots \right|\omega_{1\;N-1}\left|\omega_{3\;N}\right|\ldots \right]=\left[\ldots \left|\left(a_{i}+2b_{i}\right)\omega_{i}\right|\ldots \right]\;\left(\in \;\mathrm{group}\;B_{2}\right)’.$$

With regard to the identity morphisms, we have

(56) $$\begin{array}{l} ‘1_{X1}=1_{Y1}=1_{Z1}=1_{\mathrm{Zs}1}=\left[n_{1}\left|n_{2}\right|n_{3}\left|\ldots \right|n_{i}\left|\ldots \right|n_{N-1}\left|n_{N}\right|\ldots \right],1_{X2}=1_{Y2}=1_{Z2}\\ \;=1_{\mathrm{Zs}2}=[\omega_{0\;1}\left|\omega_{0\;2}\right|\omega_{0\;3}\left|\ldots \right|\omega_{0\;i}\left|\ldots \right|\omega_{0\;N-1}\left|\omega_{0\;N}\right|\ldots ]. \end{array}$$

Herein, we view ‘functor F: C_1_ → F(C_1_) (= C_2_)’ in following way [48,49].

(Details are shown in Appendix G)

Note that a similar definition like the composition of C_1_ based on the linear group and C_2_ based on rotational group is possible, being linked with ‘functor F’.

This is satisfied provided an adequate definition of ‘Functor F’ is given, and we presume that the morphisms described previously formulates a model that renders one of forms of the canonical “central dogma” proposed by Crick in 1958 [26].

In the transcription of RNA bases, the ‘RNA splicing’ process is well-known, whereby ‘intron sequences’ are excised, and ‘exon sequences’ are combined to condense effectual information for further interpretation in protein synthesis. However, as for further processing of the triplets of bases e.g., ‘ACG’ and ‘AUG’, a considerable number of models have been reported e.g., [12,18-21,33,34,50]. We refrain from pursuing this issue at present.

## Results

We added an imaginary base ‘E’ to the set of actual DNA bases, and composed group Z_5_ of basic translational operations on grid-points of a cruciform wallpaper pattern constructed of the five base letters. Moreover, using the same five letters, we integrated the wallpaper group as the combination of linear group over the horizontal line and the rotational group based on symmetries of a fivefold phasor diagram on the unit circle in the Gaussian plane. Additionally, changes in the sequences of the DNA bases are treated using set D, the set of all possible sequences of DNA bases that also contain ‘E’. Also, ‘D_j_’s are drawn as polygonal lines graphically. Moreover, by combining group Z_5_, the operators that rearrange bases of DNA sequences constitute the group B. Using these results, simple changes of sequences, insertions/deletions, and recombination of DNA bases are also treatable via a synthesis of group-theoretical operations between sets D and group B. Together with this, all results obtained for DNA pertain to RNA by replacing T with U. Using these tools, category theory-like language is introduced to describe the canonical “central dogma” that is expected to integrate DNA-based processes, although the overall profile and range of applicability is unclear at this stage. Alternatively, by introducing the manipulations ‘{ }’ and ‘< >’, operations on states of ‘E’s in ‘D_j_’s/‘R_j_’s, whether explicit or not, can be performed in parallel with the conventional description for DNA/RNA sequences.

## Discussion

The issue treated in this article is, roughly speaking, the combination of two ideas: one is the wallpaper pattern in the context of DNA sequencing ‘§1 – §7’ , and the other is the tentative development towards systematization of molecular/genetic biology in the style of some category-theory-like description ‘§8’. Essentially, the two are different topics although strongly connected. The former is an independent study on symmetry modeling of DNA sequences, whereas the latter can be re-expressed using different material as long as the basic elements can be treated within a category-theory-like model satisfying group theory-like postulates.

In this article, we considered a group/category theory-like treatment devising an expedient ‘E’, grid-point array, and group operations to move over the array. We discussed whether and how a more synthesized description can be constructed, using simplistic postulates of group. Next, we take the basics of category theory to describe processes, although this is only at a preliminary stage.

For an application of our ideas, we have chosen DNA sequencing from the perspective of not only coding sequences of DNA bases but also describing insertions/deletions of DNA sequences using a single operation that is an element of a group. The expeditious ‘E’ permits inserting and/or deleting sequences depending on the purpose. Specific notation was introduced so that vector-like DNA sequences and operations can be composed as a set and a group.

Ordinarily, a method to describe DNA sequences is often limited in scope by focusing on only one aspect such as recognizing each base sequentially (e.g., A, G, T, A, C…from ‘AGTAC…’) [13,33-35], where an operation like ‘rotation’, ‘transition’ or ‘conversion’ based on a certain solid is often used. Another focus of attention is the rules for interpretation of codons in synthesizing proteins from DNA sequences. The rules are defined to capture the specific activity from the viewpoint of group-like operations [18-22].It also enables us to treat three manipulations as one type of operation in the group, with easily-imaginable graphic displays such as Figure 4, although it is only an accessorial tool at this stage. Increasing the degree of freedom by one and integrating changes of coding and sequence recombination might yield some polysemous utility.

When inserting/deleting sequences of bases into the main DNA sequence, even if the endpoints of the base series are identified precisely, it appears that manipulations via ‘E’s are not always necessary. Nonetheless, to determine the final order of the bases in these cases, we must track base changes from one to the next (including ‘E’, even when lost or deleted). If we use the ‘E’-assisted manipulations for coding, we need only to examine the inclusions; the rest remains unchanged in order. Additionally, we assume that when any operation is performed, the position and number of ‘E’s should be fixed so that the order of any component of ‘D_j_’ or ‘B_j_’ is not changed, at least, during operations (e.g., (A.7)). The exception is specifically the insertion/deletion of ‘E’s such as in (29–34) and (36–45).

We briefly point out the notational benefits of the imaginary ‘E’s. These are three; 1) to adjust the sorts (number) of bases in DNAs and RNAs (from ‘4’ to ‘5’), thus enabling group-theoretical composition over the (two-dimensional) plane; 2) to link the notational sequences of DNAs and mRNAs in a single format that can be used in a more compact database to record and analyze genetic information; and 3) to express sequences of DNAs/RNAs as a vector in three different ways: a) with explicit ‘E’s in the vector, b) with implicit ‘E’s in the vector, and c) with all ‘E’s omitted in the vector except the trailing ‘E’s. The last offers flexibility in storing world-wide genetic data in a single set. We suggest that exhaustiveness is one of the potentialities of the model adding versatility in addressing the possibilities of certain behaviors of DNA/RNA sequences. While that might be far from practical applications at this moment, a more rigorous methodology in the near future may yield a means.Regarding style of the grid-point/cruciform/wallpaper pattern (Figure 1) in defining the group postulates, one of its advantages is that each base is surrounded by the four others. This symmetrical simplicity is absent in the linear group and the rotational group (Figures 1, 5 and 6), where the relative position of the five bases is fixed and thereby restrictive. Also, it might be crucial that the number ‘5’ is key in enabling composition of the sort provided by the wallpaper group using the cruciform, and an identity element necessary to satisfy the group postulates. Being a prime, ‘5’ will be convenient in further developments of the model exploiting algebraic structures such as rings or fields.

A similar synthesis might be possible between a modulo 7 additive rotational group based on a sevenfold phasor diagram with a space group depending upon six ‘forward/backward’, ‘up/down’ and ‘left/right’ directions. In practice, a space group is formed that consists of three orthogonal cruciforms comprising the six directions (±x, ±y, ±z) with seven elemental operations {m_u_(up), m_d_(down), m_r_(right), m_l_(left), m_f_(forward), m_b_(backward) and m_n_(no movement)}. These determine the operations of the group, which permute seven bases (prime number) or seven letter-like constituents. Analogous to the fivefold phasor diagram, we draw equispaced elements on the unit circle over the Gaussian plane; suppose ‘φ = 2π/7 (rad)’, then the set {φ_1_(= φ), φ_2_(= 2φ), φ_3_(= 3φ), φ_4_(= 4φ), φ_5_(= 5φ), φ_6_(= 6φ), φ_0_(= φ_7_ = 0)} parameterizes the rotational group [51], and both are, at least, in partial correspondence. We presume that, in extension, bringing together an n-dimensional space group (using the 2n + 1 elements associated with the ± n-directions and E) and a rotational group based on the n-fold phasor diagram on the unit circle (with 2n + 1 elements as points of the vertex of a polygon) might be possible. For this article, we have just focused on ‘n = 2’ in §1–§8.

Apart from the above, the model based on the wallpaper pattern might have a close relationship with ‘cellular automata’ [52]. Appropriate definitions of the wallpaper pattern for the five bases might find an expression between groups and cellular automata [53].

One consideration concerns whether a more integrated/synthesized style to describe biomolecular processes is possible using only simple, primitive defining rules, in particular, when describing genetic processes such as DNA transcription and RNA synthesis of proteins. Whereas the group postulates might be too restrictive to define molecular behavior, category postulates might enable such schemes to proceed because its postulates are weaker than those defining a group. If the interpretation of DNA by messenger RNA is definable within category theory, and protein synthesis is expressible within the same theory, there might be advantages in having the molecular system classified and treated in a reduced size in the database. At least, we conjecture that these ideas might be valid when clarifying impossible phenomena associated with changes of DNA sequences, resulting in reducing unnecessary, recrementitious efforts or roundabout paths that might encroach on researchers’ limited time for investigation. That issue might be avoided if the impossibility of certain themes was known beforehand. From this standpoint, we believe that a mathematical systematization (in a general and unexceptional manner) is crucially important for future molecular/genetic biology.

The limitations of the present model should be noted. First, the wallpaper pattern drawn in Figure 1 is one example of various patterns. In general, the wallpaper groups have been classified into seventeen categories [2,44-47]. There could be other types of patterns like Figure 1 and groups upon which to compose this sort of model. For instance, if we exchange all ‘A’s for all ‘C’s, and all ‘G’s for all ‘T’s in the model presented in this article, an almost equivalent model ‘§1–§8’ is constructed. Other arrangements might provide still unknown advantages that enable models like ours to be treated in a more rational manner. It remains unclear how to construct an optimal method to determine models yielding the wallpaper pattern of Figure 1 and the bijection given in Figure 3, and to develop the categories presented in Figures 7 and 8. The best positions of the five bases should be examined under a rigorous methodology.

Second, a Cartesian vector is defined as a combination of components on which operations are conducted independently. Indeed, we can perform operations on the i-th component of D_j_ of set D using the (i + 1)-th component of B_j_ by adding an ‘E’ any place before the (i-1)-th component of D_j_. This is because the components after the i-th of B_j_ shift to the right within the vector B_j_. Therefore, in appearance, the components at different positions are essentially spectators (e.g., a base ‘C_**3**_’ cannot change into either ‘A_**5**_’ or G_**5**_ by any B_j_ except via ‘E’-assisted manipulations). In this case, after the insertion of two ‘E’s between the ‘**2**’ and ‘**3**’ components, ‘C_**3+2**_(= C_**5**_)’ can become either ‘A_**5**_’ or G_**5**_’ by acting appropriately on B_j_ at C_**5**_. However, that might raise some confusion. For ‘E’-assisted operations (such as 29–34, 38, 40), the results might change according to the place number of inserted/deleted ‘E’s that yields the mis-matches between the ‘i-th’ component of ‘D_j_’ and that of ‘B_j_’. We believe further studies are warranted to find a descriptive format for the model.

Third, as for the graphical displays of ‘D’s in Figure 4, although the sequences of ‘D_m_’s (m = 1, 2, 3) are in reality the same, the respective expressions are not always unique because the presence of the imaginary ‘E’s changes the shape of each sequence; e.g.,‘D_1_ = [A_1_C_2_C_3_( )G_4_T_5_**E**_**6**_**E**_**7**_**E**_**8**_…]’ and ‘D_2_ = [A_1_C_2_C_3_(**E**_**4**_**E**_**5**_)G_6_T_7_**E**_**8**_**E**_**9**_**E**_**10**_…]’ are different over the wallpaper pattern despite being equivalent as real sequences. Although by use of electronic tools, these graphics might be of versatility for detection or identification of DNA sequences, these might produce other confusions in the present form. We hope that more appropriate devices would be performed in future study.

Fourth, DNA transcription to RNA and/or mRNA and translation of RNA and/or mRNA into proteins at the ribosomes are performed using a grammar rule based on a three-base set called a ‘codon’. Codons have information to synthesize twenty types of proteins; for example, ‘CAG’ codes for ‘glutamine’. As mentioned before, a number of approaches have been proposed exploit group-theoretic methods. These cover the rules for composition of triplet of bases ‘XXX’, the ways of reading codons, and models to compose geometric solids such as the tetrahedron and hexahedron, [12,18-21,33,34,50]. The rules for treating this aspect (transcription and translation of DNA bases’ information) are not established in the present article. In addition, there are specific types of codon, such as ‘TAA’, ‘TGA’, and ‘TAG’, which are presently classified as ‘stop’ or ‘halt’ commands. Aside from this, there are various rules related to biogenetic activities such as DNA repair, alternative splicing, transposition, and translocation. These specific characterizations are lacking in our model, so, further improvements on this issue are desirable.

Fifth, the traditional symmetry model of DNA bases often is based on the chemical types ‘purine/pyrimidine’, ‘amino/keto’, and ‘strong/weak hydrogen binding’ using biomolecular characteristics, which often have advantages for their treatments where three-dimensional graphics aid the imagination, and `matricized’ expressions are possible [29,35,36]. In our model, we merely use a rule for complementary pairing in §4 and §5. No restriction on couplings between ‘C, A, T, G and E’ is postulated in the present article. There might occur a number of combinations where non-realistic pairings of bases (e.g., ‘A-G’, ‘C-C’, and ‘T-E’) produce futilities and wastefulness in applications. We hope that future studies can solve this problem.

Sixth, there might be too many speculative conjectures with hypothetical situations those should be used to prove scientific facts using verified methods. Thus, a more rigorous examination for a rational style with a more effective methodology is necessary.

Our model is far from a complete systematization. However, we believe that it is necessary that some principal breakthrough should be pursued if we intend to systemize a descriptive model, and that if appropriate definitions are devised, that might help to systemize biomolecular/genetic biology in a more optimized manner with greater sophistication to make a significant contribution to the field.

## Conclusions

Within the large limitations of our methodology, it is considered that there is fertile ground where variants of the symmetry model for genetic coding based upon a specific wallpaper group are constructible. By integrating the linear group and rotational group over a specific wallpaper pattern, a more integrated formulation based on a group/category theory-like description is open to exploration in applications to a number of topics from molecular/genetic biology.

## Appendix A

According to Figures 1, 3 and 6, the following relationships are confirmed straightforwardly between any bases and independently of the type of bases:

(A.1) $$\begin{array}{l} d●d=l●u=u●l=r●n=n●r=r,\\ r●r=d●l=l●d=u●n=n●u=u,\\ l●l=r●u=u●r=d●n=n●d=d,\\ u●u=r●d=d●r=l●n=n●l=l,\\ n●n=r●l=l●r=u●d=d●u=n \end{array}$$

Here the symbol ‘↔’ signifies ‘bijection’ and the meaning of ‘x[−1, 0]’ is explained in §4. Hence, operators that are regarded to effect changes from one base to another can be re-expressed as illustrated in the following examples for various types of component operations:

(A.2) $$b_{\left[E\to C\right]}=b_{\left[C\to A\right]}=b_{\left[A\to T\right]}=b_{\left[T\to G\right]}=b_{\left[G\to E\right]}=r\;\left(↔\omega_{1}\right)=x\left[1,0\right],$$

(A.3) $$b_{\left[E\to A\right]}=b_{\left[A\to G\right]}=b_{\left[G\to C\right]}=b_{\left[C\to T\right]}=b_{\left[T\to E\right]}=u\;\left(↔\omega_{2}\right)=x\left[0,1\right],$$

(A.4) $$b_{\left[E\to T\right]}=b_{\left[T\to C\right]}=b_{\left[C\to G\right]}=b_{\left[G\to A\right]}=b_{\left[A\to E\right]}=d\;\left(↔\omega_{3}\right)=x\left[0,-1\right],$$

(A.5) $$b_{\left[E\to G\right]}=b_{\left[G\to T\right]}=b_{\left[T\to A\right]}=b_{\left[A\to C\right]}=b_{\left[C\to E\right]}=l\;\left(↔\omega_{4}\right)=x\left[-1,0\right],$$

(A.6) $$b_{\left[E\to E\right]}=b_{\left[C\to C\right]}=b_{\left[A\to A\right]}=b_{\left[T\to T\right]}=b_{\left[G\to G\right]}=n\;\left(↔\omega_{0}\left(=\mathrm{no}\;\mathrm{rotation}\right)=\omega_{5}\right)=x\left[0,0\right]$$

## Appendix B

As for B,

1) Associativity: ‘(B_j_●B_k_)●B_l_ = B_j_●(B_k_●B_l_)’ holds for all positive integers j, k and l.

2) Identity: ‘B_0_ = [n_1_|n_2_|n_3_| … |n_i_| … |n_(n ‒ 1)_|n_n_|n_n + 1_|n_n + 2_|n_n + 3_| …]’ is an identity element that satisfies ‘ B_0_●B_m_ = B_m_●B_0_ = B_m_ ’. (i = 1, 2, 3, …; ‘ n_i_ (=n) ’ is an element of Z_5_ (no movement of the point P))

3) Inverses: there exists a unique ‘B_m_^−1^’ that satisfies ‘B_m_^‒ 1^●B_m_ = B_m_●B_m_^‒ 1^ = B_0_’. Actually, the components of the inverse are the inverses of each individual component.

4) Commutativity: ‘B_j_●B_k_ = B_k_●B_j_’.

5) Closure law: any ‘B_j_●B_k_’ belongs to the set B.

## Appendix C

(A.7) $$\begin{array}{l} D_{2}\circ B_{\left(2\to 3\right)}●B_{\left(3\to 4\right)}\\ =[A_{1}\left|C_{2}\right|C_{3}\left[E_{4}|E_{5}\right]G_{6}\left|T_{7}\right|A_{N}\left|E_{8}\right|E_{9}|\ldots ]\\ \circ [n_{1}\left|n_{2}\right|n_{3}\left(u_{4}|d_{5}\right)n_{6}\left|n_{7}\right|n_{8}\left|n_{9}\right|\ldots ]●[l_{1}\left|u_{2}\right|\;d_{3}\left(r_{4}|d_{5}\right)n_{6}\left|l_{7}\right|n_{8}\left|n_{9}\right|\ldots ],\\ =\left[A_{1}\circ n_{1}●l_{1}\left|C_{2}\circ n_{2}●u_{2}\right|C_{3}\circ n_{3}●d_{3}\left(E_{4}\circ u_{4}●r_{4}|E_{5}\circ d_{5}●d_{5}\right)G_{6}\circ n_{6}●n_{6}\left|T_{7}\circ n_{7}●l_{7}\right|E_{8}\circ n_{8}●n_{8}\left|E_{9}\circ n_{9}●n_{9}\right|\ldots \right]. \end{array}$$

Again, with reference to Figure 1 or Appendix B,

(A.8) $$\begin{array}{l} =\left[A_{1}\circ l_{1}\left|C_{2}\circ u_{2}\right|C_{3}\circ d_{3}\left(E_{4}\circ d_{4}|E_{5}\circ r_{5}\right)G_{6}\circ n_{6}\left|T_{7}\circ l_{7}\right|E_{8}\circ n_{8}\left|E_{9}\circ n_{9}\right|\ldots \right],\\ =\left[C_{1}\left|T_{2}\right|G_{3}\left(T_{4}|C_{5}\right)G_{6}\left|A_{7}\right|E_{8}\left|E_{9}\right|\ldots \right]=D_{4}. \end{array}$$

## Appendix D

Naturally, the series D_k_ is generated through the following sequence of operations:

$$\begin{array}{l} B_{\left(j\to k\right)}=\left[{r^{4-1}}_{1}\left|{r^{3-2}}_{2}\right|{r^{1-0}}_{3}\left|\ldots \right|{r^{4-1}}_{i}\left|\ldots \right|{r^{1-3}}_{N-1}\left|{r^{3-2}}_{N}\right|{r^{0-0}}_{N+1}\left|{r^{0-0}}_{N+2}\right|{r^{0-0}}_{N+3}|\ldots \right],\\ \;=\left[{r^{3}}_{1}\left|{r^{1}}_{2}\right|{r^{1}}_{3}\left|\ldots \right|{r^{3}}_{i}\left|\ldots \right|{r^{-2}}_{N-1}\left|{r^{1}}_{N}\right|{r^{0}}_{N+1}\left|{r^{0}}_{N+2}\right|{r^{0}}_{N+3}|\ldots \right],\\ \;=\left[{r^{3}}_{1}\left|{r^{1}}_{2}\right|{r^{1}}_{3}\left|\ldots \right|{r^{3}}_{i}\left|\ldots \right|{r^{-2+5}}_{N-1}\left|{r^{1}}_{N}\right|{r^{0}}_{N+1}\left|{r^{0}}_{N+2}\right|{r^{0}}_{N+3}|\ldots \right],\\ \;=\left[{r^{3}}_{1}\left|{r^{1}}_{2}\right|{r^{1}}_{3}\left|\ldots \right|{r^{3}}_{i}\left|\ldots \right|{r^{3}}_{N-1}\left|{r^{1}}_{N}\right|{r^{0}}_{N+1}\left|{r^{0}}_{N+2}\right|{r^{0}}_{N+3}|\ldots \right]. \end{array}$$

Then,

(A.9) $$\begin{array}{l} D_{j}\circ B_{\left(j\to k\right)}\\ =\left[C_{1}\left|A_{2}\right|E_{3}\left|\ldots \right|C_{i}\left|\ldots \right|T_{N-1}\left|A_{N}\right|E_{N+1}\left|E_{N+2}\right|E_{N+3}|\ldots \right]\circ [{r^{3}}_{1}\left|{r^{1}}_{2}\right|{r^{1}}_{3}\left|\ldots \right|{r^{3}}_{i}|\ldots \\ \ldots \left|{r^{3}}_{N-1}\right|{r^{1}}_{N}\left|{r^{0}}_{N+1}\right|{r^{0}}_{N+2}\left|{r^{0}}_{N+3}\right|\ldots ],\\ =\left[E\circ {r^{1}}_{1}\left|E\circ {r^{2}}_{2}\right|E\circ {r^{0}}_{3}\left|\ldots \right|E\circ {r^{1}}_{i}\left|\ldots \right|E\circ {r^{3}}_{N-1}\left|E\circ {r^{2}}_{N}\right|E\circ {r^{0}}_{N+1}\left|E\circ {r^{0}}_{N+2}\right|E\circ {r^{0}}_{N+3}|\ldots \right]\\ \circ \left[{r^{3}}_{1}\left|{r^{1}}_{2}\right|{r^{1}}_{3}\left|\ldots \right|{r^{3}}_{i}\left|\ldots \ldots \right|{r^{3}}_{N-1}\left|{r^{1}}_{N}\right|{r^{0}}_{N+1}\left|{r^{0}}_{N+2}\right|{r^{0}}_{N+3}|\ldots \right]\\ =[E\circ {r^{1+3}}_{1}\left|E\circ {r^{2+1}}_{2}\right|E\circ {r^{0+1}}_{3}\left|\ldots \right|E\circ {r^{1+3}}_{i}|\ldots \\ \ldots \left|E\circ {r^{3+3}}_{N-1}\right|E\circ {r^{2+1}}_{N}\left|E\circ {r^{0}}_{N+1}\right|E\circ {r^{0}}_{N+2}\left|E\circ {r^{0}}_{N+3}\right|\ldots ],\\ =[E\circ {r^{1+3}}_{1}\left|E\circ {r^{2+1}}_{2}\right|E\circ {r^{0+1}}_{3}\left|\ldots \right|E\circ {r^{1+3}}_{i}|\ldots \\ \ldots \left|E\circ {r^{3+3}}_{N-1}\right|E\circ {r^{3}}_{N}\left|E\circ {r^{0}}_{N+1}\right|E\circ {r^{0}}_{N+2}\left|E\circ {r^{0}}_{N+3}\right|\ldots ],\\ =\left[E\circ {r^{4}}_{1}\left|E\circ {r^{3}}_{2}\right|E\circ {r^{1}}_{3}\left|\ldots \right|E\circ {r^{4}}_{i}\left|\ldots \right|E\circ {r^{6}}_{N-1}\left|E\circ {r^{3}}_{N}\right|E\circ {r^{0}}_{N+1}\left|E\circ {r^{0}}_{N+2}\right|E\circ {r^{0}}_{N+3}|\ldots \right],\\ =\left[E\circ {r^{4}}_{1}\left|E\circ {r^{3}}_{2}\right|E\circ {r^{1}}_{3}\left|\ldots \right|E\circ {r^{4}}_{i}\left|\ldots \right|E\circ {r^{6-5}}_{N-1}\left|E\circ {r^{3}}_{N}\right|E\circ {r^{0}}_{N+1}\left|E\circ {r^{0}}_{N+2}\right|E\circ {r^{0}}_{N+3}|\ldots \right],\\ =\left[E\circ {r^{4}}_{1}\left|E\circ {r^{3}}_{2}\right|E\circ {r^{1}}_{3}\left|\ldots \right|E\circ {r^{4}}_{i}\left|\ldots \right|E\circ {r^{1}}_{N-1}\left|E\circ {r^{3}}_{N}\right|E\circ {r^{0}}_{N+1}\left|E\circ {r^{0}}_{N+2}\right|E\circ {r^{0}}_{N+3}|\ldots \right],\\ =\left[G_{1}\left|T_{2}\right|C_{3}\left|\ldots \right|G_{i}\left|\ldots \right|C_{N-1}\left|T_{N}\right|E_{N+1}\left|E_{N+2}\right|E_{N+3}|\ldots \right]=D_{k}. \end{array}$$

## Appendix E

(A.10) $$\begin{array}{l} D_{j}\circ B_{\left(j\to j†\right)}\\ =[E\circ x[0,1]{}_{1}|E\circ x[0,-1]{}_{2}|E\circ x[0,0]{}_{3}\left|\ldots \right|E\circ x[1,0]{}_{i}|\ldots \\ \ldots \left|E\circ x{\left[-1,0\right]}_{N-1}\right|E\circ x{\left[0,1\right]}_{N}\left|E\circ x{\left[0,0\right]}_{N+1}\right|E\circ x{\left[0,0\right]}_{N+2}\left|E\circ x{\left[0,0\right]}_{N+3}\right|\ldots ]\\ \circ [x[0,-2]{}_{1}|x[0,2]{}_{2}|x[0,0]{}_{3}\left|\ldots \right|x[-2,0]{}_{i}\left|\ldots \right|x[2,0]{}_{N-1}|\;x[0,-2]{}_{N}\\ \left|x{\left[0,0\right]}_{N+1}\right|x{\left[0,0\right]}_{N+2}\left|x{\left[0,0\right]}_{N+3}\right|\ldots ],\\ =[E\circ r^{0}●{u^{1}}_{1}\left|E\circ r^{0}●{u^{-1}}_{2}\right|\mathrm{E\circ}\circ r^{0}●{u^{0}}_{3}\left|\ldots \right|\mathrm{E\circ}\circ r^{1}●{u^{0}}_{i}|\ldots \\ \ldots \left|E\circ r^{-1}●{u^{0}}_{N-1}\right|E\circ r^{0}●{u^{1}}_{N}\left|E\circ r^{0}●{u^{0}}_{N+1}\right|E\circ r^{0}●{u^{0}}_{N+2}\left|E\circ r^{0}●{u^{0}}_{N+3}\right|\ldots ],\\ \circ \left[r^{0}●{u^{-2}}_{1}\left|r^{0}●{u^{2}}_{2}\right|r^{0}●{u^{0}}_{3}\left|\ldots \right|r^{-2}●{u^{0}}_{i}\left|\ldots \right|r^{2}●{u^{0}}_{N-1}\left|r^{0}●{u^{-2}}_{N}\right|r^{0}●{u^{0}}_{N+1}\left|r^{0}●{u^{0}}_{N+2}\right|r^{0}●{u^{0}}_{N+3}|\ldots \right],\\ =[E\circ r^{0}●u^{1}●r^{0}●{u^{-2}}_{1}\left|E\circ r^{0}●u^{-1}●r^{0}●{u^{2}}_{2}\right|E\circ r^{0}●u^{0}●r^{0}●{u^{0}}_{3}\left|\ldots \right|E\circ r^{1}●u^{0}●r^{-2}●{u^{0}}_{i}|\ldots \\ \left|E\circ r^{-1}●u^{0}●r^{2}●{u^{0}}_{N-1}\right|E\circ r^{0}●u^{1}●r^{0}●{u^{-2}}_{N}\left|E\circ r^{0}●u^{0}●r^{0}●{u^{0}}_{N+1}\right|E\circ r^{0}●u^{0}●r^{0}●{u^{0}}_{N+2}\left|E\circ r^{0}●u^{0}●r^{0}●{u^{0}}_{N+3}\right|\ldots ],\\ =[E\circ r^{0}●{u^{-1}}_{1}\left|E\circ r^{0}●{u^{1}}_{2}\right|E\circ r^{0}●{u^{0}}_{3}\left|\ldots \right|E\circ r^{-1}●{u^{0}}_{i}|\ldots \\ \ldots \left|E\circ r^{1}●{u^{0}}_{N-1}\right|E\circ r^{0}●{u^{-1}}_{N}\left|E\circ r^{0}●{u^{0}}_{N+1}\right|E\circ r^{0}●{u^{0}}_{N+2}\left|E\circ r^{0}●{u^{0}}_{N+3}\right|\ldots ],\\ =[E\circ r^{0}●{d^{1}}_{1}\left|E\circ r^{0}●{u^{1}}_{2}\right|E\circ r^{0}●{u^{0}}_{3}\left|\ldots \right|E\circ l^{1}●{u^{0}}_{i}|\ldots \\ \ldots \left|E\circ r^{1}●{u^{0}}_{N-1}\right|E\circ r^{0}●{d^{1}}_{N}\left|E\circ r^{0}●{u^{0}}_{N+1}\right|E\circ r^{0}●{u^{0}}_{N+2}\left|E\circ r^{0}●{u^{0}}_{N+3}\right|\ldots ],\\ =\left[T_{1}\left|A_{2}\right|E_{3}\left|\ldots \right|G_{i}\left|\ldots \right|C_{n-1}\left|T_{n}\right|E_{n+1}\left|E_{n+2}\right|E_{n+3}|\ldots \right]\\ ={D_{j}}^{†}. \end{array}$$

## Appendix F

The axioms are:

(A.11) $$‘1_{X}=1_{Y}=1_{Z}=1_{\mathrm{Zs}}=\left[n_{1}\left|n_{2}\right|n_{3}\left|\ldots \right|n_{i}\left|\ldots \right|n_{N-1}\left|n_{N}\right|\ldots \right]’\;\mathrm{satisfies}\;\mathrm{these}\;\mathrm{conditions}.$$

Ι) A binary operation and closure law: the combination of two morphisms satisfies hom(X, X) × hom(X, Y) → hom(X, Y). Moreover, hom(X, Y) × hom(Y, Z) → mor (X, Z) and hom(Y, Z) × hom(Z, Zs) → mor (Y, Zs) both hold.

II) Associativity: If f: X → X, ρ: X → Y, τ: Y → Z, g: X → Z, h: Z → Z, and j: Z → Zs. Then, ‘f●(ρ●τ) = (f●ρ)●τ’, ‘ρ●(τ●h) = (ρ●τ)●h’, ‘f●(g●h) = (f●g)●h’, and ‘τ●(h●j) = (τ●h)●j’ hold.

III) Identity: there exist morphisms ‘1_X_, 1_Y_, 1_Z_, 1_Zs_’ such that ‘1_X_●f = f = f●1_X_’, and ‘1_Y_●ρ = ρ = ρ●1_X_’, ‘1_Z_●τ = τ = τ●1_Y_’, ‘1_Z_●g = g = g●1_X_’, ‘1_Z_●h = h = h●1_Z_’. ‘1_Zs_●j = j = j●1_Z_’. In practice,

## Appendix G

For Category C_1_,

(A.12) $$\begin{array}{l} \mathrm{morphism}\;f_{1}(=B_{1}\left(\in \;\mathrm{group}\;B_{1}\right)):X_{1}\to X_{1},\\ \mathrm{morphism}\;\rho_{1}:X_{1}\to Y_{1},\\ \mathrm{morphism}\;\tau_{1}:Y_{1}\to Z_{1},\\ \mathrm{morphism}\;g_{1}\left(=\rho_{1}●\tau_{1}\right):X_{1}\to Z_{1},\\ \mathrm{morphism}\;h_{1}(=B_{1}\left(\in \;\mathrm{group}\;B_{1}\right)):Z_{1}\to Z_{1},\\ \mathrm{morphism}\;j_{1}:Z_{1}\to Zs_{1}. \end{array}$$

Similarly for category C_2_, for each object F(X_1_) = X_2_, F(Y_1_) = Y_2_, F(Z_1_) = Z_2_, F(Zs_1_) = Zs_2_ (∈C_2_), the following relationships also hold:

(A.13) $$\begin{array}{l} \mathrm{morphism}\;F\left(f_{1}\right)\;(=f_{2}=B_{2}\left(\in \;\mathrm{group}\;B_{2}\right)):F\left(X_{1}\right)\to F\left(X_{1}\right),\\ \mathrm{morphism}\;F\left(\rho_{1}\right)\;\left(=\rho_{2}\right):F\left(X_{1}\right)\to F\left(Y_{1}\right),\\ \mathrm{morphism}\;F\left(\tau_{1}\right)\;\left(=\tau_{2}\right):F\left(Y_{1}\right)\to F\left(Z_{1}\right),\\ \mathrm{morphism}\;F\left(g_{1}\right)\;\left(=g_{2}=\rho_{2}●\tau_{2}\right):F\left(X_{1}\right)\to F\left(Z_{1}\right),\\ \mathrm{morphism}\;F\left(h_{1}\right)\;(=h_{2}=B_{2}\left(\in \;\mathrm{group}\;B_{2}\right)):F\left(Z_{1}\right)\to F\left(Z_{1}\right),\\ \mathrm{morphism}\;F\left(j_{1}\right)\;\left(=j_{2}\right):F\left(Z_{1}\right)\to F\left(Zs_{1}\right). \end{array}$$

Other than these, if relationships F(f_1_●ρ_1_) = F(f_1_)●F(ρ_1_), F(ρ_1_●τ_1_) = F(ρ_1_)●F(τ_1_), F(τ_1_●h_1_) = F(τ_1_)●F(h_1_), F(f_1_●g_1_) = F(f_1_)●F(g_1_), F(g_1_●h_1_) = F(g_1_)●F(h_1_), and F(h_1_●j_1_) = F(h_1_)●F(j_1_) are satisfied, the composition of C_1_ and C_2_ linked with ‘functor F’ is possible although the proof is omitted here.

Furthermore, the following postulates hold: for object X (∈C_1_), ‘F(1_X_) = 1_F(X)_ (∈C_2_)’ is true, for object Y (∈C_1_), ‘F(1_Y_) = 1_F(Y)_ (∈C_2_)’ and for object Z (∈C_1_), ‘F(1_Z_) = 1_F(Z)_ (∈C_2_)’, is true under the condition:

(A.14) $$‘F\left(1_{X}\right)=1_{F\left(X\right)}=F\left(1_{Y}\right)=1_{F\left(Y\right)}=F\left(1_{Z}\right)=1_{F\left(Z\right)}=\left[\omega_{0\;1}\left|\omega_{0\;2}\right|\omega_{0\;3}\left|\ldots \right|\omega_{0\;i}\left|\ldots \right|\omega_{0\;N-1}\left|\omega_{0\;N}\right|\ldots \right]’.$$

## Competing interests

The authors declare that they have no competing interests.

## Authors’ contributions

JS conceived of the main idea of this article and wrote the manuscript. SM revised the manuscript. JI gave advice on potential versatilities of the model to the biological science. In addition, all authors read and approved the final version of the manuscript.
